# Reduced Esterification Rather Than Increased Hydrolysis Is Causative for Loss of Hepatic Retinoids Upon CCl_4_ ‐Induced Liver Injury

**DOI:** 10.1111/liv.70213

**Published:** 2025-08-06

**Authors:** Carina Wagner, Kristina Košić, Dominik Bulfon, Johannes Breithofer, Alina Jamnik, Clara Zitta, Paula Horvat, Kim Bilweis, Michael Schupp, Robert Zimmermann, Ulrike Taschler, Achim Lass

**Affiliations:** ^1^ Institute of Molecular Biosciences, NAWI Graz, University of Graz Graz Austria; ^2^ Charité Universitätsmedizin Berlin, Corporate Member of Freie Universität Berlin and Humboldt‐Universität zu Berlin Institute of Pharmacology, Max Rubner Center (MRC) for Cardiovascular‐Metabolic‐Renal Research Berlin Germany; ^3^ BioTechMed‐Graz Graz Austria; ^4^ Field of Excellence BioHealth, University of Graz Graz Austria

**Keywords:** fibrosis, lecithin:retinol acyltransferase, retinyl ester hydrolases, vitamin A

## Abstract

**Background and Aims:**

Advanced liver disease leads to liver fibrosis that is characterised by the activation of non‐parenchymal stellate cells, accumulation of extracellular matrix proteins, and the loss of hepatic vitamin A stores. To date, the molecular mechanisms and enzymes mediating the loss of hepatic vitamin A stores are incompletely understood.

**Approach and Results:**

Using a fibrosis mouse model induced by the hepatotoxin carbon tetrachloride (CCl_4_), we investigated which cellular processes in the liver mediate the loss of hepatic retinyl ester stores. We found that repeated CCl_4_ injections into mice over six weeks led to a biphasic change in plasma retinol levels that were increased after three and decreased after six weeks as compared to control mice. As expected, livers of mice receiving CCl_4_ injections showed increased expression of pro‐fibrogenic genes that were accompanied by decreased hepatic retinoid content, which was mainly due to loss of retinoids in non‐parenchymal cells (NPCs). In the liver and NPCs, decreased retinyl ester levels correlated with reduced gene expression of lecithin:retinol acyltransferase and reduced hepatic ex vivo retinol acyltransferase activity. Conversely, gene expression of lipases known to exhibit retinyl ester hydrolase activity (REHA) remained unchanged or was decreased, consistent with decreased neutral REHA in homogenates of respective livers and lysates of isolated NPCs, respectively. Albeit hepatic expression levels of marker proteins for autophagosomal and lysosomal membranes were increased, gene expression of the major acidic retinyl ester hydrolase, lysosomal acid lipase, as well as ex vivo acidic REHA were reduced in NPC lysates.

**Conclusion:**

Together, these results indicate that the loss of hepatic retinyl ester stores upon liver injury and stellate cell activation is rather a consequence of reduced retinol esterification than neutral or acidic hydrolysis.

**Summary**

- Chronic CCl_4_‐induced liver injury in mice leads to fibrogenesis, the activation of hepatic stellate cells, and the loss of hepatic retinoid stores.
- Isolated non‐parenchymal cells, but much less hepatocytes from livers of CCl_4_‐treated mice show decreased retinoid content, indicating that the loss mostly occurs in the retinoid‐storing hepatic stellate cells.
- Mechanistically, we show that the loss of retinoids upon CCl_4_‐induced liver injury is caused by decreased retinol esterification due to downregulation of lecithin:retinol acyltransferase rather than increased retinyl ester hydrolysis by neutral or acidic hydrolases.

**Abbreviations**
α‐SMA: alpha‐smooth muscle actin
Abhd5: alpha/beta hydrolase domain containing 5
Adh1/4: alcohol dehydrogenase 1/4
Aldh1a1: aldehyde dehydrogenase 1 family member A1
ApoB: apolipoprotein B
ATG7: autophagy related 7
ATGL: adipose triglyceride lipase
BAT: brown adipose tissue
BSA: bovine serum albumin
CCl_4_
: carbon tetrachloride
Ces: carboxylesterase
CGI‐58: comparative gene identification‐58
Col1a1: procollagen type 1a1
Col1a2: procollagen type 1a2
CRBP: cellular retinol binding protein
CS: coomassie stain
CycloB: cyclophilin B
Cyp26a1: cytochrome P450 family 26 subfamily A member 1
Dgat1: diacylglycerol O‐acyltransferase 1
FD: fluorescence detection
G0S2: G0/G1 switch gene 2
GAPDH: glyceraldehyde‐3‐phosphate dehydrogenase
HSL: hormone‐sensitive lipase
LAL: lysosomal acid lipase
LAMP1: lysosomal‐associated membrane protein 1
LC‐3B: microtubule‐associated protein 1A/1B‐light chain 3B
Ldlr: low‐density lipoprotein receptor
Lipe: lipase E
Lox: lysyl oxidase
LRAT: lecithin:retinol acyltransferase
Lrp1: low‐density lipoprotein related protein 1
Mttp: microsomal triglyceride transfer protein
Nceh1: neutral cholesterol ester hydrolase 1
NPC: non‐parenchymal cell
p62: sequestosome 1
PGAT: perigonadal adipose tissue
PNPLA2/3: patatin‐like phospholipase domain‐containing protein 2/3
RAB7: ras‐related protein 7
Rarβ: retinoic acid receptor beta
RBP4: retinol binding protein 4
SCAT: subcutaneous adipose tissue
Tgfß: transforming growth factor beta
Timp1: TIMP metallopeptidase inhibitor 1

## 1 Introduction

Chronic liver injury eventually progresses to liver fibrosis, a scarring process replacing damaged tissue with extracellular matrix proteins. A hallmark in the onset of liver fibrosis is the activation of specialised non‐parenchymal liver cells, the hepatic stellate cells [1, 2]. In a healthy liver, hepatic stellate cells reside in a quiescent state and store large quantities of vitamin A in form of retinyl esters that ensure a constant supply under times of nutritional vitamin A undersupply. Because of their high lipid content, hepatic stellate cells have also been denoted as vitamin A‐storing cells, lipocytes, fat‐storing cells, or Ito cells [3, 4, 5, 6]. In response to liver injury, hepatic stellate cells trans‐differentiate from quiescent vitamin A‐storing into activated fibrogenic, myofibroblast‐like cells. Activated hepatic stellate cells proliferate and express extracellular matrix proteins and enzymes for remodelling thereof [7]. Importantly, in the process of hepatic stellate cell activation and transformation, cells lose their ability to store vitamin A [8]. Consequently, hepatic vitamin A stores are lost and thus low hepatic vitamin A content often correlates with the progression of liver disease [9, 10, 11, 12].

Vitamin A (retinol and derivatives) is an essential micronutrient that has to be obtained from diet. In liver, vitamin A is stored in form of retinyl esters in cytosolic lipid droplets of parenchymal hepatocytes but to a much larger extent in non‐parenchymal hepatic stellate cells, which comprise around 80%–90% of total hepatic retinyl ester content [13]. The storage of hepatic retinyl esters is mainly balanced by opposing processes: (i) The cellular uptake of vitamin A‐containing lipoproteins from the circulation and the subsequent esterification of retinol by the action of lecithin:retinol acyltransferase (LRAT) promoting retinyl ester storage. This storage is counteracted by (ii) the breakdown of retinyl esters by hydrolases and the release of retinol:RBP4 complexes into the circulation [14, 15]. Two different pathways have been described to facilitate the breakdown of retinyl ester stores: First, the degradation of lipid droplet‐contained retinyl esters by neutral retinyl ester hydrolases, including lipases such as adipose triglyceride lipase (ATGL), hormone‐sensitive lipase (HSL), patatin‐like phospholipase domain‐containing protein 3 (PNPLA3), KIAA1363, and members of the carboxylesterase (Ces) family [15, 16, 17, 18, 19, 20, 21]. Second, the breakdown of entire retinyl ester‐containing lipid droplets by lipophagy, involving the engulfment of lipid droplets by autophagosomes, fusion with lysosomes, and the subsequent degradation of retinyl esters by lysosomal acidic lipase (LAL) in autolysosomes [22, 23, 24, 25]. To date, however, the functional role and mechanisms underlying the loss of hepatic vitamin A stores during the development of liver fibrosis are poorly understood.

## 2 Materials and Methods

### 2.1 Materials

Essentially fatty acid‐free bovine serum albumin (BSA), retinyl palmitate, retinyl acetate, 1,2‐dioleoyl‐*sn*‐glycero‐3‐phosphocholine, L‐α‐phosphatidylinositol sodium salt, collagenase type II, and pronase E were purchased from Sigma‐Aldrich (St. Louis, Missouri). Lalistat2 was a kind gift from Dr. Paul Helquist (Department of Chemistry and Biochemistry, University of Notre Dame, Notre Dame, Indiana).

### 2.2 Animals

C57Bl/6J mice were housed on a regular light–dark cycle (14 h light, 10 h dark) at 22°C ± 1°C in a specific pathogen‐free environment and had *ad libitum* access to food and water. Mice were kept on a standard laboratory chow diet (R/M‐H Extrudate, V1126‐027, 25 IU/g vitamin A, Ssniff Spezialdiaeten GmbH, Soest, Germany). Blood samples were collected by orbital venous sinus bleeding and EDTA plasma was prepared. *Ad libitum* fed mice were anaesthetised with isoflurane and euthanised by cervical dislocation. Tissues were dissected, flash‐frozen in liquid nitrogen, and stored at −80°C until further use. All animal experiments were approved by the Austrian Federal Ministry for Science, Research, and Economy (protocol number GZ: 39/9/75 ex 2017/18), and conducted in compliance with the Council of Europe Convention (ETS 123).

### 2.3 Induction of Hepatic Stellate Cell Activation and Liver Fibrosis

In order to induce chronic liver fibrosis, male C57Bl/6J mice received i.p. injections of CCl_4_ (0.6 μL/g body weight, 1:4 diluted in corn oil) or vehicle (corn oil) as control twice per week over a period of 6 weeks. Blood samples were collected prior to the treatment and after 3 and 6 weeks of CCl_4_ administration. Mice were sacrificed and tissues were collected 2 days after the last CCl_4_ injection, in the *ad libitum* fed state at an age of 16 weeks.

### 2.4 Isolation of Hepatocyte and Non‐Parenchymal Cell (NPC) Fractions by Collagenase Perfusion

Hepatocyte‐ and NPC‐enriched fractions from vehicle‐ and CCl_4_‐treated mice were isolated as described previously by Blomhoff et al. [26] with some modifications. Briefly, mice were anaesthetised and the abdomen was surgically opened. The liver was perfused via the portal vein with Krebs–Henseleit buffer (without Ca^2+^ and SO_4_^2−^) for 5 min, followed by a perfusion with Krebs–Henseleit buffer containing 0.3 mg/mL collagenase type II, 0.2 mg/mL pronase E, 2% BSA, and 0.1 mM CaCl_2_ for 10 min. Afterwards, the liver was excised, minced, and the tissue suspension was passed through a gauze, followed by filtration through a 70 μm cell strainer. Hepatocytes were separated from NPCs by centrifugation at 50 × *g* for 3 min at 4°C. Supernatant containing the NPC fraction was centrifuged at 900 × *g* for 5 min at 4°C. Pelleted hepatocytes and NPCs were washed twice with phosphate‐buffered saline and snap‐frozen in liquid nitrogen.

### 2.5 Extraction and Quantification of Retinoids by HPLC‐Fluorescence Detection (FD)

Retinoid extraction was performed as follows: briefly, cell or tissue samples were homogenised in 200 μL phosphate‐buffered saline, 200 μL ethanol (containing 3.5 μM retinyl acetate as internal standard), and 1 mL *n*‐hexane (containing 1 mM butylhydroxytoluol) using a ball mill (Retsch GmbH, Haan, Germany). Phase separation was achieved by centrifugation at 5000 × *g* at 4°C for 10 min, and the upper organic phase was collected. For repeated extraction, 1 mL *n*‐hexane was added to the remaining tissue/cell homogenate and vigorously vortexed for 20 s. For phase separation, samples were again centrifuged at 5000 × *g* at 4°C for 10 min. Organic phases were combined and dried in a speed‐vac (Labconco Corp., Kansas City, MO). Retinoids of plasma samples (20 μL) were directly *n*‐hexane extracted by vortexing as described above. For retinoid analyses by HPLC‐FD, retinoid extracts were dissolved in methanol:toluene (1:1, v/v), and separated on a YMC‐Pro C18 column (150 × 4.6 mm, S‐3 μL, 12 nm, YMC Europe GmbH, Dinslaken, Germany) using a gradient solvent system (flow, 2 mL/min, gradient: 0–2 min 100% methanol, 2–4.2 min 60%/40% methanol/toluene, and 4.2–6 min 100% methanol). Fluorescence was detected at excitation 325 nm/emission 490 nm. The HPLC consisted of a Waters e2695 separation module, a column oven (at 25°C), and a Waters 2475 fluorescence detector (Waters Corp., Milford, MA). Data were analysed using Empower 3 chromatography data software (Waters Corp.). Area under the peak was standardised against known amount of the internal standard retinyl acetate. Retinoid content was normalised to whole tissue weight.

### 2.6 Extraction and Quantification of Bis(Monoacylglycero)Phosphate and Triglyceride by LC‐ESI‐MS

Lipids were extracted according to Matyash et al. [27] with modifications using 0.7 mL MTBE/MeOH (3:1; v/v) containing 500 pmol butylhydroxytoluol, 0.01% acetic acid, and internal standard (150 pmol 14:0–14:0 bis(monoacylglycero)phosphate, Avanti Polar Lipids, Alabaster, Alabama and 40 pmol 17:0–17:0–17:0 triglyceride, Larodan, Solna, Sweden) per sample. Samples were extracted under constant shaking on a thermomixer at 1200 rpm for 30 min at room temperature. After the addition of 150 μL ddH_2_O and further incubation for 10 min at room temperature, samples were centrifuged for phase separation at 20 000 × *g* for 5 min at room temperature. Fivehundred μl of upper organic phase were collected, dried under a stream of nitrogen, and reconstituted in MeOH/2‐propanol/ddH_2_O (6:3:1; v/v/v) for UPLC/MS analysis. An Agilent 1290 Infinity II UHPLC, fitted with an ACQUITY UPLC BEH C18 Column 2.1 × 150 mm, 1.7 μm (Waters Corp.), was used for chromatographic separation. The column temperature was set to 50°C, and the flow rate was set to 0.2 mL/min. MeOH/H_2_O (8:2, v/v) made up mobile phase A, whereas 2‐propanol/MeOH (8:2, v/v) made up mobile phase B. Both mobile phases contained 10 mM ammonium acetate, 0.1% formic acid, and 8 μM phosphoric acid. The chromatography was a 30 min gradient that opened with a linear increase in mobile phase B from 50% to 60% during a 13 min period. This was followed by a 7 min linear gradient to 100% mobile phase B, which was maintained for 5 min before dropping back to 50%. Bis(monoacylglycero)phosphate and triglyceride species were detected on an Agilent 6470 triple‐quadrupole mass spectrometer with Agilent Jet Stream ESI (Agilent Technologies, Santa Clara, California) as previously described [28]. Data processing was performed with Agilent MassHunter quantitative analysis software version 10.1 and Agilent MassHunter qualitative analysis software version 10.0. Data were corrected for recovery, extraction, and ionisation efficacy by calculating analyte/internal standard ratios (AU), and normalised to tissue or protein content (AU/g tissue or AU/mg protein).

### 2.7 Preparation of Liver Lysates and Determination of Protein Content

Tissues or cells were homogenised in solution A (0.25 M sucrose, 1 mM dithiothreitol, 1 mM EDTA, pH 7.0), containing protease inhibitor (20 μg/mL leupeptin, 2 μg/mL antipain, 1 μg/mL pepstatin). Tissues were homogenised using an Ultra‐Turrax homogeniser (T10 basic Ultra‐Turrax, IKA, Staufen, Germany). Cell lysates were prepared by sonication (2 × 10 s, amplitude 15%; Sonoplus ultrasonic homogeniser HD3100, Bandelin electronic GmbH & Co. KG, Berlin, Germany). Liver or cell homogenates were centrifuged at 1000 × *g* for 10 min at 4°C, and 1000 × *g* supernatants were collected. In some cases, 1000 × *g* supernatants were centrifuged at 100 000 × *g* for 1 h at 4°C, and 100 000 × *g* supernatants were collected. Protein concentrations were determined by Bio‐Rad protein assay (Bio‐Rad, Hercules, California) according to the manufacturer's instructions using BSA as standard.

### 2.8 Determination of Ex Vivo Retinyl Ester Hydrolase Activity

Determination of ex vivo retinyl ester hydrolase activity was performed as previously described [21]. Briefly, 100 μL liver or cell lysates (1000 × *g* supernatant containing 50–100 μg protein) were incubated with 100 μL retinyl palmitate (300 μM) as substrate at 37°C for 1 h under constant shaking in a water bath. Retinyl palmitate was emulsified with phosphatidyl choline (300 μM) or CHAPS (20 mM) in potassium phosphate buffer (100 mM, pH 7.5) or potassium acetate buffer (100 mM, pH 4.5) containing 4% fatty acid‐free BSA, respectively. In some cases, liver or cell lysates contained DMSO as solvent control or the LAL‐specific inhibitor Lalistat2 (20 μM). Substrate blank incubation was performed with solution A. Retinoid extraction and quantification were performed as described above. In some cases, fatty acid release was determined using Fujifilm NEFA‐HR Assay (Wako Chemicals Europe GmbH, Neuss, Germany) according to the manufacturer's instructions.

### 2.9 Determination of Ex Vivo Retinyl Ester Formation Activity

In order to determine ex vivo retinyl ester formation activity, liver lysates (100 000 × *g* supernatant containing 100 μg protein) were incubated with Expi293F cell lysates containing murine cellular retinol binding protein (CRBP, 50 μg protein) and 100 μL retinol (900 μM) as substrate for 1 h at 37°C. Retinol was emulsified with phosphatidyl choline (400 μM) in potassium phosphate buffer (100 mM, pH 7.5) containing 0.5% fatty acid‐free BSA. To distinguish between LRAT and acyl CoA:retinol acyltransferase activity, some liver lysates contained 1 mM PMSF, known to inhibit LRAT activity [29], or DMSO as solvent control. Substrate blank incubation was performed with solution A. Retinoid extraction and quantification were performed as described above. The values for the activity of PMSF‐incubated cell lysates were subtracted from DMSO‐incubated cell lysates.

### 2.10 Analysis of Protein Expression by Immunoblotting

Tissue lysates (5–20 μg) were dissolved in SDS‐PAGE sample buffer and proteins were denatured at 95°C for 10 min. Proteins were separated by 10% or 12.5% SDS‐PAGE and transferred onto a polyvinylidene difluoride membrane (Carl Roth GmbH, Karlsruhe, Germany). The membrane was blocked with 10% non‐fat dry milk for 1 h and incubated with the respective primary antibodies: anti‐ATGL, anti‐HSL, anti‐GAPDH, anti‐ATG7, anti‐p62, anti‐LC3B, anti‐LAMP1, anti‐RAB7, anti‐mTOR, and anti‐phospho‐mTOR (Ser2448) from Cell Signalling Technology (Danvers, MA; 2138S/ATGL, 4107S/HSL, 2118S/GAPDH, 8558S/ATG7, 5114S/p62, 2775S/LC3B, C54H11/LAMP1, 2094S/RAB7, 2972S/mTOR, 2971S/phoshpo‐mTOR), anti‐RBP4 from DAKO (Agilent Technologies, A0040), anti‐CGI‐58 from Abnova (Taipei, Taiwan, H00051099‐M01), anti‐ApoB from Abcam (Cambridge, United Kingdom, ab20737), anti‐Vinculin from Sigma‐Aldrich (V9131), and anti‐α‐SMA from Thermo Fisher Scientific (Waltham, Massachusetts, PA5‐22251), respectively. For detection, membranes were incubated with horseradish peroxidase‐labelled secondary antibodies specific for the respective primary antibody. Bands were visualised using the ECL plus Western blotting Detection Reagent (Thermo Fisher Scientific) and ChemiDoc Touch Imaging System (Bio‐Rad, Hercules, California).

### 2.11 Isolation of Total RNA and Gene Expression Analysis by qPCR

RNA from liver or cell samples was extracted as described previously [24]. Livers (~50 mg) were homogenised in 0.5 mL TRIzol (Invitrogen GmbH, Waltham, Massachusetts) using an Ultra‐Turrax homogeniser, followed by a 5 min incubation at room temperature. Hepatocytes and NPCs were directly harvested in TRIzol. Phase separation was achieved by the addition of 100 μL 1‐bromo‐3‐chlorpropane/mL TRIzol and centrifugation at 12 000 × *g* and 4°C for 15 min. Supernatant was transferred and total RNA was precipitated with 0.5 mL 2‐propanol/ml TRIzol and pelleted by centrifugation at 12 000 × *g* and 4°C for 10 min. RNA pellets were washed twice with 70% ethanol and centrifuged at 10 000 × *g* and 4°C for 5 min. RNA was digested with DNaseI and reverse‐transcribed into cDNA using LunaScript RT SuperMix kit (New England Biolabs Inc., Ipswich, Massachusetts). qPCR was performed using StepOnePlus Real‐Time PCR system (Applied Biosystems, Waltham, Massachusetts) with denaturation at 95°C and annealing/extension at 60°C for 40 cycles. PCR reactions (20 μL) contained 8 ng cDNA, 10 pmol of forward and reverse primer, and 10 μL SYBR Green Master Mix (Bio‐Rad). Primers used for qPCR are listed in Table S1. Target gene expression was calculated by the ΔΔCT method. Expression of the housekeeping gene cyclophilin B (*CycloB*) was used for normalisation.

### 2.12 Statistical Analyses

Data are presented as mean + standard deviation (S.D.). Statistically significant differences were determined by Student's unpaired *t*‐test (two‐tailed). Group differences were considered statistically significant for *p* < 0.05 (*), *p* < 0.01 (**), *p* < 0.001 (***).

## 3 Results

### 3.1 Prolonged Administration of CCl_4_ to C57Bl/6J Mice Results in Initially Increased, Followed by Reduced Levels of Plasma Retinol and RBP4

**FIGURE 1:**
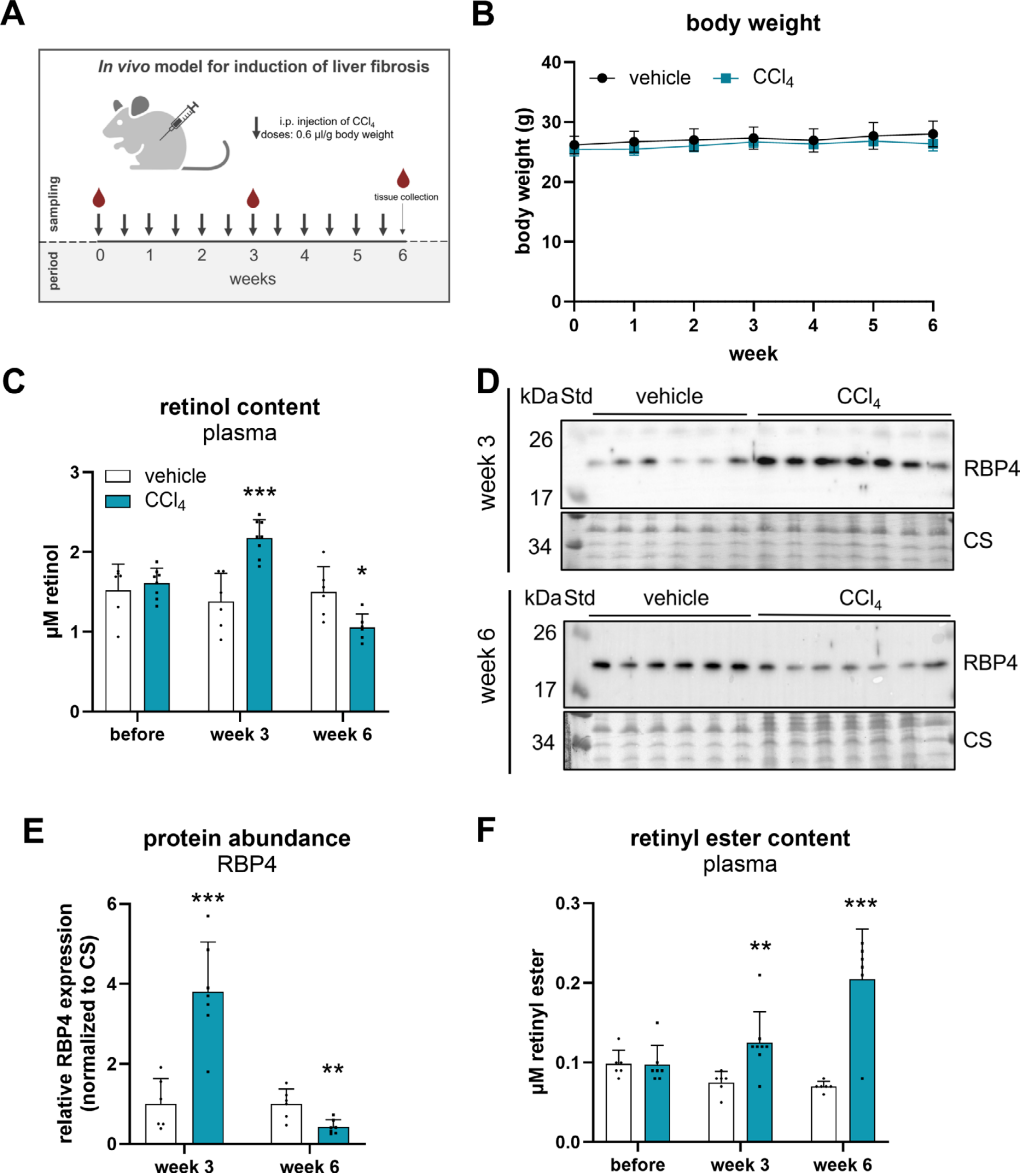
Circulating retinol, retinyl ester, and RBP4 levels are altered upon CCl_4_ treatment in *ad libitum* fed C57Bl/6J mice. (A) Schematic representation of the experimental setup. To induce liver fibrosis, CCl_4_ (0.6 μL/g body weight, diluted in corn oil, *n* = 7) and corn oil as vehicle control (*n* = 6) were administered (i.p.) to C57Bl/6J mice twice a week for 6 weeks. Blood samples were collected in the *ad libitum* fed state as indicated. Tissues were harvested in the *ad libitum* fed state 2 days *post* last CCl_4_ injection. (B) Body weight of vehicle‐ and CCl_4_‐treated mice was recorded weekly. Plasma of vehicle‐ and CCl_4_‐treated mice was *n*‐hexane extracted and (C) retinol and (F) retinyl ester levels were analysed by HPLC‐FD. (D) Protein abundance of RBP4 after 3 and 6 weeks of CCl_4_ administration was analysed by Western blotting. Coomassie stain (CS) was used as loading control. (E) Band intensities corresponding to RBP4 were quantified using Bio‐Rad Image Lab Software. Data are mean + S.D. and represented as individual data points of biological replicates. Statistically significant differences were determined by Student's unpaired *t*‐test (two‐tailed; *p < 0.05, **p < 0.01, ***p < 0.001).

To study the effect of prolonged liver injury on hepatic and whole‐body vitamin A homeostasis, we repeatedly treated C57Bl/6J mice with the hepatotoxin CCl_4_ to induce liver damage and hepatic stellate cell activation. As depicted in the schematic representation of the experimental setup (Figure 1A), mice received 12 injections of CCl_4_ (0.6 μL/g body weight) or corn oil (vehicle control) over 6 weeks. Blood samples were collected before the treatment, as well as after 3 and 6 weeks of treatment in the *ad libitum* fed state. Tissues were collected in the *ad libitum* fed state after 6 weeks of treatment, 2 days *post* last CCl_4_ injection. Body weights of vehicle‐ and CCl_4_‐treated mice were recorded weekly and remained virtually unchanged over the 6 weeks as well as between treatment groups (Figure 1B). Further, we also assessed longitudinal changes in plasma retinoid and RBP4 levels. Vehicle‐treated control mice showed unchanged plasma retinol levels at all time points measured (Figure 1C). In contrast, mice receiving CCl_4_ injections exhibited a 60% increase and a 30% decrease in plasma retinol levels after 3 and 6 weeks of treatment, respectively, as compared to vehicle control mice (Figure 1C). In accordance, plasma RBP4 levels showed a similar pattern to retinol levels and were 3.8‐fold increased and 40% decreased after 3 and 6 weeks of CCl_4_ treatment (Figure 1D,E), respectively, as compared to controls. Interestingly, we observed that after 3 and 6 weeks of CCl_4_ treatment, plasma retinyl ester levels of CCl_4_‐treated mice were 1.7‐ and 3‐fold higher as compared to control mice (Figure 1F).

These findings collectively suggest that in the initial phase of liver injury, plasma retinol and RBP4 levels were elevated as compared to control mice. However, upon prolonged CCl_4_ treatment, these levels decreased, suggesting biphasic changes in circulating plasma retinol and RBP4 levels. Moreover, the observation of increased plasma retinyl ester levels suggests prolonged circulation of lipoproteins/chylomicrons in the plasma of mice upon the manifestation of liver damage.

### 3.2 CCl_4_ Treatment of C57Bl/6J Mice Induces Hepatic Stellate Cell Activation, Accompanied by Reduced Hepatic Retinyl Ester Content

**FIGURE 2:**
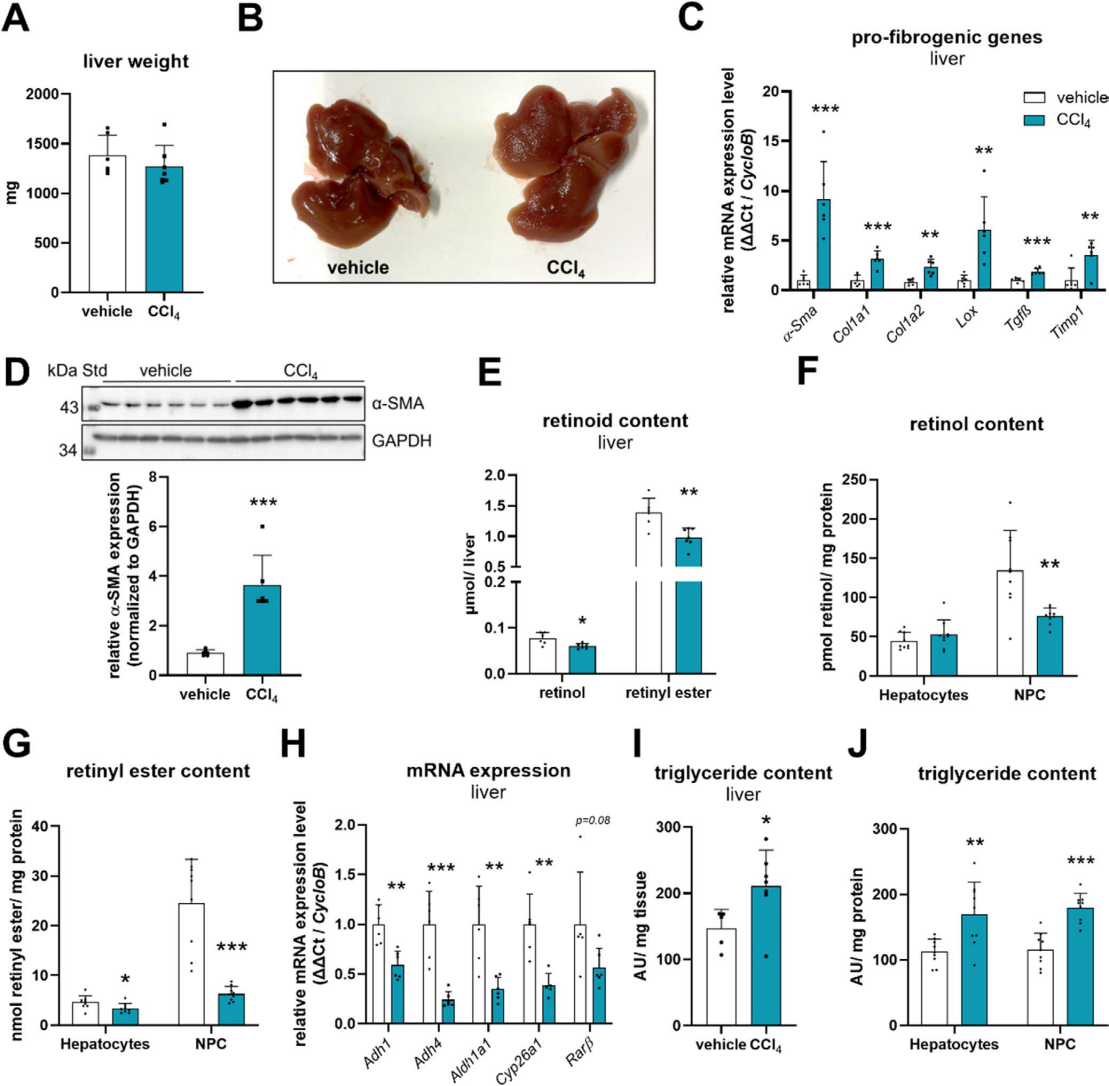
Hepatic retinyl ester levels are lower in CCl_4_‐ as compared to vehicle‐treated *ad libitum* fed C57Bl/6J mice. CCl_4_ (0.6 μL/g body weight, diluted in corn oil, *n* = 7–10) or vehicle (corn oil, *n* = 6–9) was administered (i.p.) to C57Bl/6J mice twice a week for 6 weeks. Tissues were harvested in the *ad libitum* fed state 2 days *post* last CCl_4_ injection. Hepatocyte and NPC fractions were isolated by liver perfusion and differential centrifugation. (A) Liver weights of vehicle‐ and CCl_4_‐treated mice. (B) Images of livers isolated from *ad libitum* fed vehicle‐ (left) and CCl_4_‐treated (right) mice. Isolated RNA from livers of vehicle‐ and CCl_4_‐treated mice was transcribed and gene expression of (C) *α‐Sma*, *Col1a1*, *Col1a2*, *Lox*, *Tgfβ*, *Timp1*, (H) *Adh1*, *Adh4*, *Aldh1a1*, *Cyp26a1*, *Rarβ*, and *CycloB* was determined by qPCR. (D) Protein expression of α‐SMA in livers of vehicle‐ and CCl_4_‐treated mice was analysed by Western blotting. GAPDH expression was used as loading control. Band intensities corresponding to α‐SMA and GAPDH expression were quantified using Bio‐Rad Image Lab software. (E–G) Livers as well as isolated hepatocytes and NPCs were *n*‐hexane extracted, and retinol and retinyl ester levels were analysed by HPLC‐FD. Retinoid content was normalised to whole liver weight (E) or mg protein (F, G). Triglyceride levels of livers (I) as well as isolated hepatocytes and NPCs (J) were determined by LC–MS. Triglyceride content was calculated based on the sum of signals of detected molecular species. Data are mean + S.D. and represented as individual data points of biological replicates. Statistically significant differences were determined by Student's unpaired *t*‐test (two‐tailed; *p < 0.05, **p < 0.01, ***p < 0.001).

Next, we examined livers of C57Bl/6J mice to evaluate for signs of CCl_4_‐induced liver injury. After 6 weeks of CCl_4_ administration, livers of *ad libitum* fed CCl_4_‐treated mice showed similar tissue weights as compared to vehicle‐treated control mice (Figure 2A). Macroscopic examination of livers obtained from CCl_4_‐treated mice showed coarse parenchymal nodules on the surface of livers that were not seen on livers from vehicle‐treated control mice (Figure 2B). These obvious changes in the texture of the livers from CCl_4_‐treated mice were accompanied by increased expression of the stellate cell activation marker alpha‐smooth muscle actin (α‐SMA) at mRNA (Figure 2C) as well as protein levels (Figure 2D). Similarly, mRNA expression of pro‐fibrogenic genes such as procollagen type 1 (*Col1a1* and *Col1a2*), lysyl oxidase (*Lox*), transforming growth factor beta (*Tgfβ*), and TIMP metallopeptidase inhibitor 1 (*Timp1*) was several‐fold increased (3‐, 2.5‐, 6‐, 2‐, 3.5‐fold, respectively) (Figure 2C). Nodular appearance of liver surface as well as increased expression of fibrosis markers are consistent with hepatic stellate cell activation as well as the manifestation of liver fibrosis after 6 weeks of CCl_4_ administration in C57Bl/6J mice.

Liver damage in CCl_4_‐treated mice was associated with a 22% and 30% decrease in hepatic retinol and retinyl ester content, respectively, as compared to control mice (Figure 2E). To investigate which hepatic cell type accounts for the reduction of hepatic retinoids upon liver injury, we isolated hepatocytes and NPCs of livers from vehicle‐ and CCl_4_‐treated mice by collagenase perfusion, the latter cell fraction also containing the vitamin A‐storing hepatic stellate cells. We observed that hepatocytes of CCl_4_‐treated mice contained unchanged retinol levels while retinyl ester content declined by 26% (Figure 2F,G). In the NPC fraction, retinoids were prominently reduced and contained 43% and 74% less retinol and retinyl ester levels, as compared to controls (Figure 2F,G). This indicates that reduced hepatic retinoid content, in particular reduced retinyl ester levels, was largely due to decreased retinyl ester content of non‐parenchymal hepatic stellate cells. Reduced hepatic retinol levels were accompanied by reduced mRNA expression of the retinol‐oxidising enzymes alcohol dehydrogenases 1 and 4 (*Adh1*, *Adh4*). Moreover, the retinoid‐metabolising and retinoic acid‐responsive genes aldehyde dehydrogenase 1 family member A1 (*Aldh1a1*) [30] and cytochrome P450 family 26 subfamily A member 1 (*Cyp26a1*) [31] were decreased by more than 60% in livers of CCl_4_‐treated mice (Figure 2H). Consistently, the retinoic acid‐responsive gene retinoic acid receptor beta (*Rarβ*, Figure 2H) [32] showed tentatively reduced expression. For comparison, we also measured triglyceride content in whole liver as well as isolated hepatocyte and NPC fractions obtained from vehicle‐ or CCl_4_‐treated mice. In whole liver samples of CCl_4_‐treated mice, triglycerides were elevated 1.7‐fold (Figure 2I) as compared to controls. A similar accumulation of triglycerides was observed in the hepatocyte‐ and NPC‐enriched fractions isolated from livers of CCl_4_‐treated mice (Figure 2J), suggesting that CCl_4_ treatment of mice led to impaired hepatic triglyceride turnover.

Together, decreased hepatic retinoid levels, which were mainly due to a loss of retinoids in the NPC fraction, and reduced gene expression of retinoid‐metabolising enzymes and retinoic acid responsive genes suggest reduced retinoic acid signalling and metabolisation.

### 3.3 CCl_4_ Treatment Leads to Reduced Hepatic LRAT Expression and Activity in C57Bl/6J Mice

**FIGURE 3:**
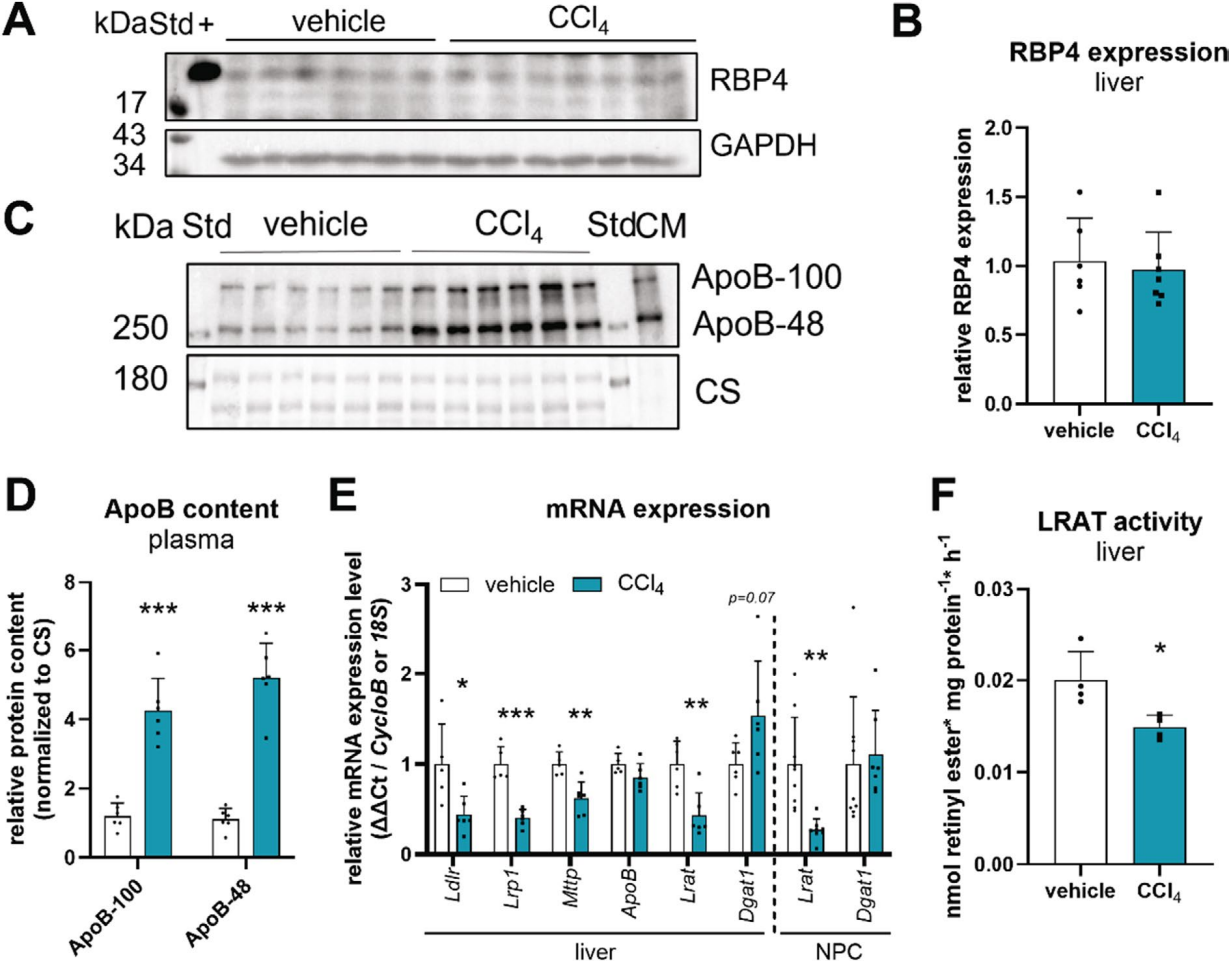
CCl_4_ treatment leads to reduced LRAT expression and activity in livers of *ad libitum* fed C57Bl/6J mice. CCl_4_ (0.6 μL/g body weight, diluted in corn oil, *n* = 7–10) or vehicle (corn oil, *n* = 6–9) was administered (i.p.) to C57Bl/6J mice twice a week for 6 weeks. Tissues were harvested in the *ad libitum* fed state 2 days *post* last CCl_4_ injection. Hepatocyte and NPC fractions were isolated by liver perfusion and differential centrifugation. Protein levels of (A) RBP4 in liver and (C) ApoB in plasma of vehicle‐ and CCl_4_‐treated mice were analysed by Western blotting. GAPDH expression or Coomassie stain (CS) were used as loading controls, respectively. (A) Plasma sample of an *ad libitum* fed C57Bl/6J mouse (+) or (C) a chylomicron‐enriched fraction (CM) isolated from plasma of refed high fat diet‐fed C57Bl/6J mice were used as positive control for RBP4 or ApoB expression, respectively. Band intensities corresponding to (B) RBP4 and (D) ApoB expression were quantified using Bio‐Rad Image Lab software. (E) Isolated mRNA from livers and NPC fractions of vehicle‐ and CCl_4_‐treated mice was transcribed, and gene expression of *Ldlr*, *Lrp1*, *Mttp*, *ApoB*, *Lrat*, *Dgat1*, *CycloB*, and *18S rRNA* was determined by qPCR. (F) For ex vivo LRAT activity assays, liver lysates (100 000 × *g* supernatant) were incubated with cell lysates containing murine CRBP (50 μg protein) and retinol (900 μM) as substrate for 1 h at 37°C. Retinol was emulsified with phosphatidyl choline (400 μM) in potassium phosphate buffer (100 mM, pH 7.5) containing 0.5% fatty acid‐free BSA. Retinol esterification activity was analysed by HPLC‐FD. Activity assays were performed in quadruple determinations. To distinguish between LRAT (inhibited by 1 mM PMSF) and acyl CoA:retinol acyltransferase activity, values for the activity of PMSF‐incubated cell lysates were subtracted from those obtained from DMSO‐incubated cell lysates. Data are mean + S.D. and represented as individual data points of biological replicates. Statistically significant differences were determined by Student's unpaired *t*‐test (two‐tailed; *p < 0.05, **p < 0.01, ***p < 0.001).

To explore factors contributing to decreased hepatic retinol and retinyl ester levels, we first analysed hepatic expression levels of RBP4, required for retinol secretion into the circulation. Interestingly, protein expression of RBP4 in livers of CCl_4_‐treated mice was unchanged as compared to controls (Figure 3A,B), implicating that hepatic retinol secretion into the circulation remained unchanged.

The observation of increased plasma retinyl ester levels in CCl_4_‐treated mice (Figure 1F) suggests prolonged circulation of lipoproteins as a result of reduced hepatic uptake that may translate into reduced hepatic retinol and retinyl ester levels. Thus, we next assessed plasma levels of the chylomicron marker apolipoprotein B‐48 (ApoB‐48) and the VLDL marker ApoB‐100 as well as hepatic mRNA expression of low‐density lipoprotein receptor (*Ldlr*) and low‐density lipoprotein related protein 1 (*Lrp1*) [33]. Indeed, we observed a 4‐ and 5‐fold increase in circulating levels of ApoB‐48 and ApoB‐100, respectively, in plasma of CCl_4_‐treated mice compared to controls (Figure 3C,D). In accordance, we observed a 56% and 60% downregulation of *Ldlr* and *Lrp1* gene expression, respectively (Figure 3E), consistent with reduced plasma clearance of lipoprotein particles. Since we observed a higher expression of the VLDL marker ApoB‐100 in plasma of CCl_4_‐treated mice (Figure 3C,D), we next assessed hepatic gene expression of the microsomal triglyceride transfer protein (*Mttp*) and *ApoB*, both markers for the assembly and secretion of VLDLs from liver. While mRNA expression of *Mttp* was 38% reduced in livers of CCl_4_‐treated mice compared to controls, *ApoB* mRNA expression remained unchanged (Figure 3E), indicating that the hepatic assembly and secretion of VLDLs was, if at all, rather decreased upon liver injury. Together, these observations suggest that reduced hepatic retinoid levels in CCl_4_‐treated mice may be a result of reduced clearance of circulating lipoprotein particles and concomitantly reduced hepatic uptake of lipoprotein‐derived retinyl esters. Further, reduced expression of *Mttp*, indicative of lower lipoprotein assembly and secretion, would be in line with increased hepatic triglyceride levels.

Another factor contributing to reduced hepatic retinyl ester levels of CCl_4_‐treated mice might be the downregulation of the retinol esterifying enzyme LRAT, an expected consequence of hepatic stellate cell activation [34]. In accordance with induced expression of pro‐fibrogenic genes indicative of stellate cell activation (Figure 2C), we observed a 57% decrease in hepatic *Lrat* mRNA expression in CCl_4_‐treated mice as compared to controls (Figure 3E). An even more pronounced reduction in *Lrat* mRNA expression (by 73%) was observed in isolated NPCs from CCl_4_‐treated mice (Figure 3E). In comparison, the gene expression of diacylglycerol O‐acyltransferase 1 (*Dgat1*), catalysing the generation of triglycerides and, to a lesser extent, of retinyl esters (acyl‐CoA:retinol acyltransferase activity [35]), was slightly affected or unchanged in livers and isolated NPCs of CCl_4_‐treated mice, respectively (Figure 3E). To assess whether the decrease in *Lrat* expression translated into reduced hepatic retinyl ester formation activity, we performed ex vivo LRAT‐dependent retinol esterification activity assays using liver lysates from vehicle‐ and CCl_4_‐treated mice. LRAT activity assays showed a 24% reduction of ex vivo retinol esterification activity in liver lysates from CCl_4_‐treated mice as compared to controls (Figure 3F). This indicates that reduced hepatic retinyl ester levels in fibrotic livers are a direct consequence of decreased *Lrat* expression and retinol esterification activity.

### 3.4 Neutral Ex Vivo Retinyl Ester Hydrolase Activity Is Decreased in Fibrotic Livers of C57Bl/6J Mice

**FIGURE 4:**
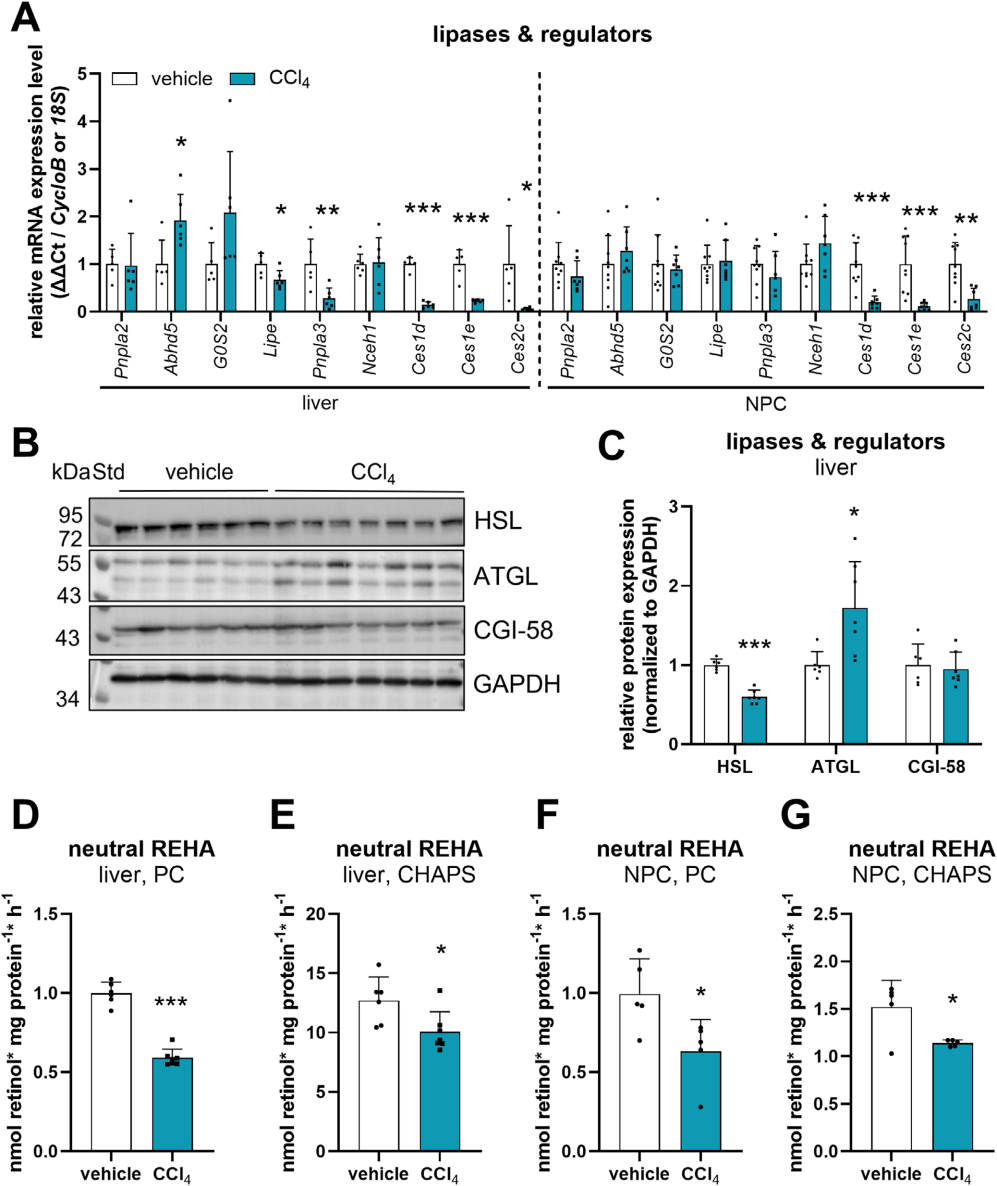
Neutral hepatic ex vivo retinyl ester hydrolase activity (REHA) is decreased in CCl_4_‐ compared to vehicle‐treated *ad libitum* fed C57Bl/6J mice. CCl_4_ (0.6 μL/g body weight, diluted in corn oil, *n* = 7–10) or vehicle (corn oil, *n* = 6–9) was administered (i.p.) to C57Bl/6J mice twice a week for 6 weeks. NPC fractions were isolated by liver perfusion and differential centrifugation. (A) Isolated mRNA from livers and NPC fractions of vehicle‐ and CCl_4_‐treated mice was transcribed, and gene expression of *Pnpla2*, *Abhd5*, *G0S2*, *Lipe*, *Pnpla3*, *Nceh1*, *Ces1d*, *Ces1e*, *Ces2c*, and *CycloB* was determined by qPCR. (B) Protein expression of HSL, ATGL, and CGI‐58 was analysed by Western blotting. GAPDH expression was used as loading control. (C) Band intensities corresponding to HSL, ATGL, CGI‐58, and GAPDH expression were quantified using Bio‐Rad Image Lab software. (D–G) For hepatic ex vivo REHA assay, liver and NPC lysates (1000 × *g* supernatant) were incubated with retinyl palmitate (300 μM) as substrate for 1 h at 37°C. Retinyl palmitate was emulsified with (D, F) phosphatidyl choline (PC, 300 μM) or (E, G) CHAPS (20 mM) in potassium phosphate buffer (100 mM, pH 7.5) containing 4% fatty acid‐free BSA. Activity assays were performed in duplicates. Data are mean + S.D. and represented as individual data points of biological replicates. Statistically significant differences were determined by Student's unpaired *t*‐test (two‐tailed; *p < 0.05, **p < 0.01, ***p < 0.001).

To examine whether increased hydrolysis of retinyl esters contributes to the observed loss of hepatic retinoid stores in livers of CCl_4_‐treated mice, we next investigated the expression of several established neutral retinyl ester hydrolases and regulators [15, 16, 17, 18, 19, 21]. Expression of *Pnpla2* (=ATGL) was unchanged on mRNA (Figure 4A), but 1.7‐fold increased on protein level in livers of CCl_4_‐treated mice compared to livers of vehicle‐treated mice (Figure 4B,C). The expression of *Abhd5* (=CGI‐58), the coactivator of ATGL, was 2‐fold increased on mRNA level in livers of CCl_4_‐treated mice (Figure 4A), but was unchanged on protein level (Figure 4B,C). The hepatic gene expression of *G0S2*, the inhibitory protein of ATGL, remained unchanged (Figure 4A). In contrast to ATGL expression, *Lipe* (=HSL) and *Pnpla3* gene expression were decreased by 33% and 72%, respectively. In line, HSL protein expression in CCl_4_‐treated mice was reduced in livers (Figure 4B,C). Furthermore, in livers of CCl_4_‐treated mice, the gene expression of *Nceh1* (also known as KIAA1363) remained unaltered, and the expression of members of the *Ces* family, *Ces1d*, *Ces1e*, and *Ces2c*, known to exhibit retinyl ester hydrolase activity [15, 20], was reduced by 85%, 77%, and 94%, respectively, as compared to vehicle‐treated mice (Figure 4A). Similar to what was observed in livers of CCl_4_‐treated mice, gene expression of *Ces1d*, *Ces1e*, and *Ces2c* was strongly decreased in isolated NPCs, while that of *Pnpla2*, *Abhd5*, *G0S2*, *Lipe*, *Pnpla3*, and *Nceh1* was virtually unchanged as compared to controls (Figure 4A).

To determine whether these changes in the expression pattern of lipases and regulators affected hepatic retinyl ester hydrolase activity, we performed ex vivo retinyl ester hydrolase activity assays at neutral pH using liver lysates of respective mice. Using retinyl ester substrate emulsified with phosphatidyl choline to mimic cytosolic lipid droplets, we observed a decline in retinol release (Figure 4D), indicative of reduced ex vivo retinyl ester hydrolase activity. We also performed similar ex vivo activity assays by emulsifying the retinyl ester substrate with CHAPS, a detergent frequently used for measuring endoplasmic reticulum‐associated enzyme activity, and observed a 25% and 33% decrease in retinol (Figure 4F) and fatty acid release (Figure S1A), respectively. A similar reduction of the retinyl ester hydrolase activity (37% and 25%, respectively) was observed when we incubated lysates of the NPC fractions from CCl_4_‐treated mice with retinyl palmitate emulsified with phosphatidyl choline (Figure 4E) or CHAPS (Figure 4G), respectively. Additionally, we performed triglyceride hydrolase activity assays at neutral pH with respective liver lysates, but did not observe differences between the treatment groups (Figure S1B).

Together, livers and isolated NPCs of CCl_4_‐treated mice showed, in most cases, an unchanged or downregulated expression of lipases known to exhibit retinyl ester hydrolase activity that was accompanied by decreased ex vivo neutral retinyl ester hydrolase activity, but unchanged neutral triglyceride hydrolase activity, in respective liver and NPC lysates. These observations indicate that upon CCl_4_‐induced liver injury, hepatic retinyl ester hydrolase activity is decreased, which correlates with reduced hepatic retinol levels and is unlikely to be causative for reduced hepatic retinyl ester levels.

### 3.5 Markers for Autolysosomal Membranes but Not Acid Hydrolytic Activity Are Increased in Livers of CCl_4_ ‐Treated C57Bl/6J Mice

**FIGURE 5:**
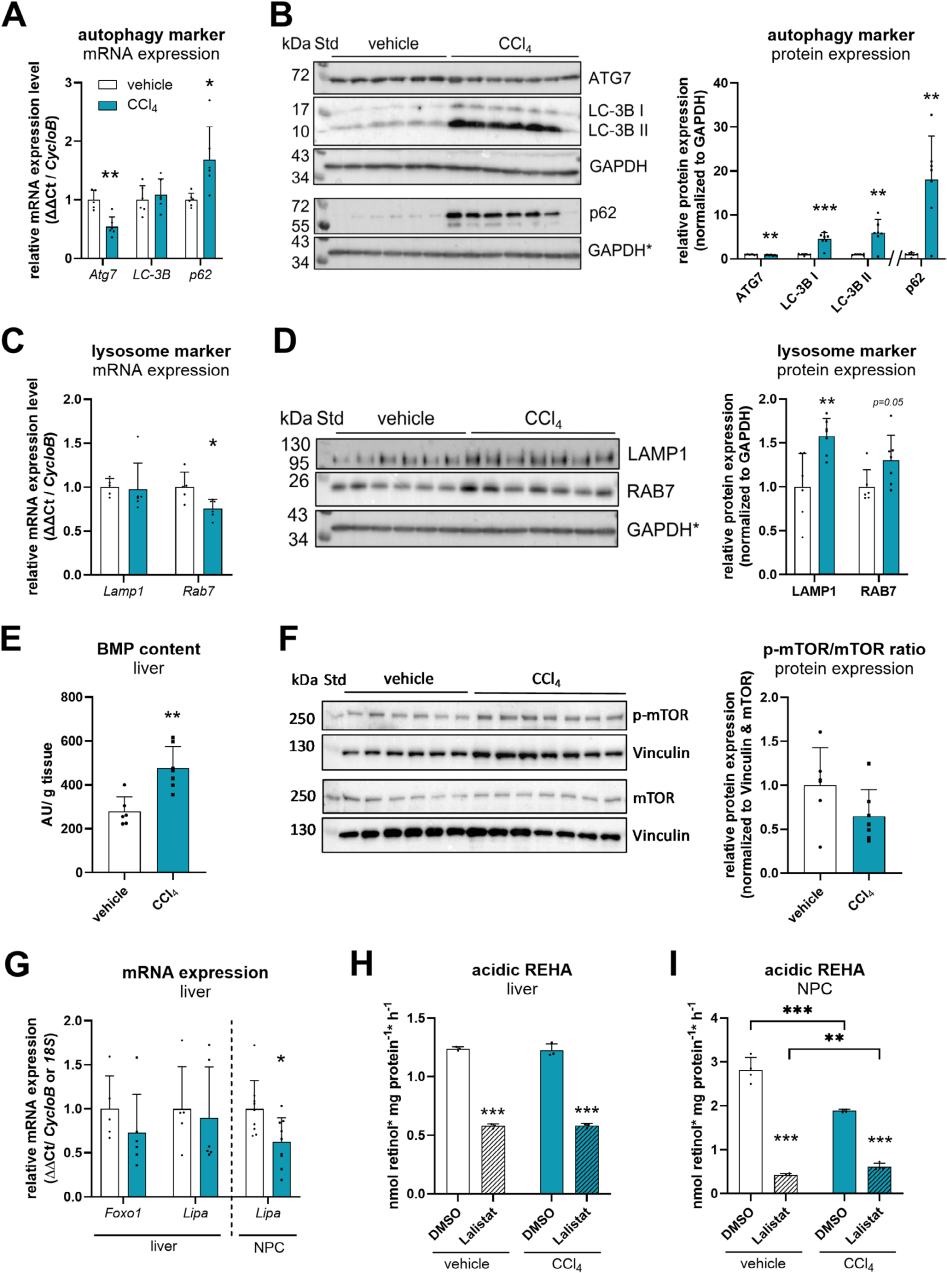
Autophagosomal and lysosomal membrane markers are increased, but acidic retinyl ester hydrolase activity (REHA) is unaltered in CCl_4_‐ compared to vehicle‐treated *ad libitum* fed C57Bl/6J mice. CCl_4_ (0.6 μL/g body weight, diluted in corn oil, *n* = 7–10) or vehicle (corn oil, *n* = 6–9) was administered (i.p.) to C57Bl/6J mice twice a week for 6 weeks. NPC fractions were isolated by liver perfusion and differential centrifugation. Isolated mRNA from livers or NPC fractions of vehicle‐ and CCl_4_‐treated mice was transcribed, and gene expression of (A) *Atg7*, *LC‐3B*, *p62*, (C) *Lamp1*, *Rab7*, (G) *Foxo1*, *Lipa*, *18S rRNA*, and *CycloB* was determined by qPCR. Protein expression of (B) ATG7, LC‐3B, p62, (D) LAMP1, RAB7, (F) p‐mTOR, and mTOR was analysed by Western blotting. GAPDH or Vinculin expression was used as loading control, respectively. *LAMP1, RAB7, and p62 detection originates from the same membrane, and therefore the same GAPDH control is shown. Band intensities corresponding to (B) ATG7, LC‐3B, p62, (D) LAMP1, RAB7, (F) p‐mTOR, mTOR, and GAPDH or Vinculin expression were quantified using Bio‐Rad Image Lab software. (E) Livers of vehicle‐ and CCl_4_‐treated mice were MTBE‐extracted, and bis(monoacylglycero)phosphate (BMP) content was analysed by mass spectrometry. Total BMP content represents the sum of analysed subspecies. (H, I) For hepatic ex vivo REHA, liver and NPC lysates (1000 × *g* supernatant) were incubated with retinyl palmitate (300 μM) as substrate for 1 h at 37°C. Retinyl palmitate was emulsified with phosphatidyl choline (300 μM) in potassium acetate buffer (100 mM, pH 4.5) containing 4% fatty acid‐free BSA. Lalistat 2 (LALi, 20 μM) or DMSO as solvent control were added to liver lysates. Activity assay was performed in triplicates. Data are mean + S.D. and represented as individual data points of biological replicates. Statistically significant differences were determined by Student's unpaired *t*‐test (two‐tailed; *p < 0.05, **p < 0.01, ***p < 0.001).

Autophagy has been shown to be involved in the degradation of lipid droplets during hepatic stellate cell activation [36]. Furthermore, several studies demonstrated the induction of autophagosome formation upon CCl_4_‐induced liver damage in isolated murine hepatic stellate cells [37, 38] and whole liver tissue [39, 40, 41, 42]. Thus, we investigated the induction of autophagosome formation in liver tissue of our chronic CCl_4_‐treated mice since this is more accessible than isolated hepatic stellate cells that would require cultivation for several days [43]. First, we analysed gene and protein levels of different autophagy and lysosome marker proteins by qPCR and Western blotting. We found that gene and protein expression of autophagy related gene 7 (ATG7), involved in autophagophore formation, was decreased in livers of CCl_4_‐treated mice compared to vehicle‐treated mice (Figure 5A,B). Moreover, hepatic mRNA expression of microtubule‐associated protein 1A/1B‐light chain 3B (*LC‐3B*) was unaffected between vehicle‐ and CCl_4_‐treated mice (Figure 5A). However, protein levels of the cytosolic form of LC‐3B, LC‐3B I and the phosphatidylethanolamine conjugate LC‐3B II, indicative of autophagosome assembly, were many‐fold increased in livers of CCl_4_‐treated mice (Figure 5B). Additionally, mRNA and protein levels of sequestosome 1 (p62), a measure of autophagic flux, were both increased upon CCl_4_ treatment (Figure 5A,B). Hepatic gene expression of lysosomal‐associated membrane protein 1 (*Lamp1*), a marker for lysosomal membranes [44], and of Ras‐related protein 7 (*Rab7*), a key regulator of autophagosome‐lysosome fusion [45] and lipophagy [46], were unchanged or decreased upon CCl_4_ treatment, respectively (Figure 5C). However, protein levels of LAMP1 and RAB7 were rather increased in livers of CCl_4_‐treated mice compared to controls (Figure 5D). Elevated protein levels of LC‐3B II and LAMP1 indicated increased autophagosomal/lysosomal content that was in line with accumulation of the lysosomal signature lipid bis(monoacylglycero)phosphate in livers of CCl_4_‐treated mice (Figure 5E). We also analysed the expression levels of the transcription factor forkhead box O‐1 (*Foxo‐1*) and protein phosphorylation of mammalian target of rapamycin (mTOR), known regulators of autophagy that directly and inversely correlate with induced autophagy, respectively [47, 48]. We found unchanged *Foxo‐1* gene expression (Figure 5G) and phospho‐mTOR protein levels (Figure 5F), further underlining that autophagy was not induced at the regulatory level in livers of CCl_4_‐treated mice.

To investigate autolysosomal‐mediated degradation of retinyl esters in the acidic compartment more directly, we analysed hepatic expression and activity of LAL, the main acid retinyl ester hydrolase in lysosomes [24]. We found that *Lipa* (=LAL) expression was comparable in livers from CCl_4_‐ and vehicle‐treated mice, but 38% decreased in NPC fractions obtained from livers of CCl_4_‐treated mice (Figure 5G). Acidic ex vivo retinyl ester hydrolase activity in liver lysates of CCl_4_‐treated mice remained unchanged as compared to controls (Figure 5H). The addition of the LAL‐specific inhibitor Lalistat 2 (LALi) reduced acidic retinyl ester hydrolase activity to a similar extent in liver lysates from mice treated with CCl_4_ and vehicle (by 50%, Figure 5H). Conversely to whole liver lysates, we observed a 33% reduced acidic ex vivo retinyl ester hydrolase activity when we used lysates of NPCs isolated from CCl_4_‐treated mice, compared to controls (Figure 5I). Moreover, the addition of LALi resulted in a prominent reduction (68% and 85%, respectively) of acidic retinyl ester hydrolase activity in liver homogenates as well as lysates of isolated NPCs (Figure 5I), in line with the notion that LAL is the major acid lipase [24]. In summary, our data indicate that LAL expression and LAL‐specific activity is rather downregulated in livers of CCl_4_‐treated mice and thus hydrolysis of retinyl esters in the acidic compartment is not induced.

Together, our data suggest that livers of CCl_4_‐treated mice show induced autophagosomal membrane formation and increased lysosome content. However, this resulted in lower *LAL* mRNA expression or LAL‐mediated acidic hydrolytic activity in NPC‐enriched fractions, suggesting that auto−/lysosomal degradation of retinyl esters was not induced.

### 3.6 Chronic CCl_4_ Treatment of C57Bl/6J Mice Leads to a Redistribution of Retinoid Stores

**FIGURE 6:**
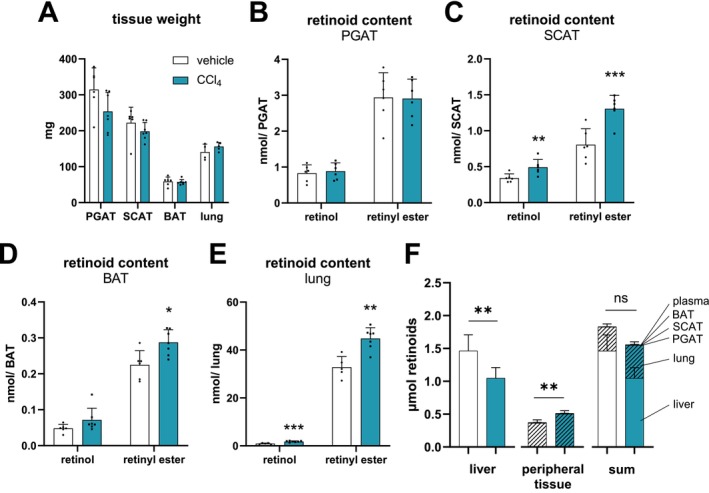
Retinoid levels in SCAT, BAT, and lung are increased in CCl_4_‐ compared to vehicle‐treated *ad libitum* fed C57Bl/6J mice. CCl_4_ (0.6 μL/g body weight, diluted in corn oil, *n* = 4–7) or vehicle (corn oil, *n* = 5–6) was administered (i.p.) to C57Bl/6J mice twice a week for 6 weeks. Tissues were harvested in the *ad libitum* fed state 2 days *post* last CCl_4_ injection. (A) Tissue weights of perigonadal adipose tissue (PGAT), subcutaneous adipose tissue (SCAT), brown adipose tissue (BAT), and lung. (B) PGAT, (C) SCAT, (D) BAT, and (E) lung, were *n*‐hexane extracted, and retinol and retinyl ester levels were analysed by HPLC‐FD. Retinoid content was normalised to whole tissue weight. (F) Retinoid content (sum of retinol and retinyl ester) from liver (from Figure 2E) and peripheral tissues, including plasma (from Figure 1C,F) (sum of PGAT, SCAT, BAT, lung, and plasma), was calculated. “Sum” represents the sum of total retinoid content from liver, peripheral tissues, and plasma. The labeling of peripheral tissues in (F) “sum” CCl_4_ also applies to the “vehicle” group (but is not indicated). Data are mean + S.D. and represented as individual data points of biological replicates. Statistically significant differences were determined by Student's unpaired *t*‐test (two‐tailed; *p < 0.05, **p < 0.01, ***p < 0.001). ns—not significant.

To explore the consequences of the manifestation of liver fibrosis and the concomitant changes in retinoid homeostasis of peripheral tissues, we analysed retinol and retinyl ester levels of different adipose tissue depots, including perigonadal adipose tissue (PGAT), subcutaneous adipose tissue (SCAT), and brown adipose tissue (BAT), as well as lung. Tissue weights of PGAT, SCAT, BAT, and lung remained unchanged in CCl_4_‐treated mice as compared to control mice (Figure 6A). While retinol and retinyl ester levels were unchanged in PGAT depots (Figure 6B), retinol and retinyl ester levels were 1.4‐ and 1.6‐fold increased in SCAT depots obtained from CCl_4_‐treated compared to vehicle‐treated mice (Figure 6C). Similarly, retinyl ester levels were 1.3‐fold higher in BAT depots of CCl_4_‐treated mice (Figure 6D). The most pronounced increase in retinoid levels upon CCl_4_ treatment was observed in lung, resulting in 1.9‐ and 1.4‐fold increased retinol and retinyl ester levels, respectively (Figure 6E).

We next asked whether the observed changes in tissue retinoid stores upon induction of liver fibrosis led to a net loss of retinoids or a redistribution between tissues in mice. Therefore, we calculated total retinoid stores of liver (=sum of retinol, retinyl palmitate, retinyl stearate, and retinyl oleate; Figure 6F see “liver”), the sum of total retinoid content of all analysed peripheral tissues including plasma (= sum of total retinoids of SCAT, PGAT, BAT, lung, and plasma; Figure 6F see “peripheral tissues”), as well as the sum of liver, peripheral tissues, and plasma retinoids (Figure 6F see “sum”) of mice treated with vehicle or CCl_4_. Calculations of the total retinoid content of tissues revealed a drop in total retinoid stores in liver by 0.44 +/− 0.32 μmol and an increase of total retinoids in peripheral tissues by 0.21 +/− 0.15 μmol of mice treated with CCl_4_ (Figure 6F, different tissues are labelled for the CCl_4_‐treated mice but are similarly also applicable for the vehicle control mice, although not indicated). This drop in liver retinoids and concomitant increase in retinoids of peripheral tissues results in overall unchanged whole‐body retinoid stores in CCl_4_‐treated mice, compared to controls (Figure 6F). These calculations indicate that, although we only assessed retinoid content of some of the tissues, whole‐body retinoid stores were rather redistributed from liver to peripheral tissue than lost via excretion in our CCl_4_‐induced liver fibrosis model.

## 4 Discussion

The loss of hepatic vitamin A stores is a concomitant effect of advanced liver disease and marks the onset of the scar‐forming process, hepatic fibrosis [49]. To date, the molecular mechanisms and enzymes responsible for the loss of hepatic vitamin A stores are incompletely understood. Therefore, we aimed to investigate the mechanisms driving the loss of hepatic vitamin A stores in a mouse model of liver fibrosis, induced by repeated injections of the hepatotoxin CCl_4_. In this study, we focused on the regulation of enzymes that facilitate either retinol esterification or retinyl ester hydrolysis, and the processes of autolysosomal degradation of entire retinyl ester stores. However, based on our observations we will also discuss other mechanisms that may contribute to altered hepatic retinyl ester stores.

A notable observation when measuring plasma retinoid levels was that repeated injections of CCl_4_ into mice led to increased plasma retinyl ester and increased plasma lipoprotein ApoB‐100 and ApoB‐48 levels, which were associated with decreased hepatic *Ldlr* and *Lrp1* gene expressions. These changes suggest that CCl_4_‐induced liver damage led to impaired hepatic lipoprotein uptake and, as a consequence, to prolonged circulation of lipoproteins. This interpretation is in line with our preliminary observation of unchanged gene expression of lipoprotein lipase in adipose tissue of CCl_4_‐treated mice (data not shown), since lipoprotein lipase is the rate‐limiting enzyme in the clearance of circulating lipoproteins [50]. If lipoprotein lipase expression were decreased, this would lead to increased plasma lipoprotein and retinyl ester levels [51, 52]. Furthermore, hepatocytes are known to internalise the majority (~70%) of circulating retinyl esters contained in chylomicron remnants by receptor‐mediated uptake [53]. These mostly chylomicron‐derived retinyl esters are then transferred to hepatic stellate cells, the principal storage site for vitamin A in the liver [54, 55], and stored in lipid droplets. Thus, it is conceivable that prolonged circulation of lipoproteins due to decreased hepatic uptake will affect hepatic retinyl ester levels. The fact that the total amount of differentially increased plasma retinyl ester levels of 0.25 nmoles (estimating a blood volume of 2.5 mL per mouse) between CCl_4_‐treated and control mice is much smaller than the loss of hepatic retinyl ester content of roughly 500 nmoles in livers of CCl_4_‐treated mice renders a prolonged circulation of lipoproteins unlikely as the primary cause for the difference in hepatic retinyl ester content. However, since we have not determined hepatic uptake of lipoproteins per se or studied the flux of retinyl esters from circulation to liver over time, the contribution of prolonged circulation/reduced hepatic uptake may be more significant than is apparent from the pure calculations. Along the line of reduced hepatic uptake of vitamin A, several human studies have reported that nutritional deficits often occur in patients with chronic liver diseases [56, 57, 58, 59, 60]. Thereby, hepatic retinoid content is affected by reduced nutritional retinoid uptake due to a cholestatic liver and decreased intralumenal bile salt concentrations, leading to impaired absorption of vitamin A [61]. Inadequate food intake per se can also contribute to lower hepatic vitamin A storage but has not been assessed in this study [62].

LRAT is the key enzyme responsible for retinyl ester formation in the liver [14] with the highest expression in non‐parenchymal hepatic stellate cells [18, 63]. Notably, upon stellate cell activation, LRAT expression is known to be downregulated [34, 64, 65]. In accordance, we also observed, in addition to increased expression of hepatic fibrogenic markers indicative of stellate cell activation, reduced *Lrat* gene expression in livers and isolated NPCs of CCl_4_‐treated mice. In line, this was accompanied by reduced retinol esterification activity in respective liver homogenates and lysates of isolated NPCs. Our observations are in accordance with similar studies in rats and mice, employing single or repeated injections of CCl_4_, as a model for liver fibrosis, reporting reduced *Lrat* gene expression that associates with increased fibrogenic markers [34, 66]. However, in other studies, no difference in hepatic *Lrat* gene expression was observed after acute or chronic CCl_4_ treatment of mice, while in rats chronic injections of CCl_4_ resulted in increased hepatic *Lrat* gene expression [67, 68]. Moreover, Kluwe et al. reported, in addition to reduced LRAT expression levels in whole livers of CCl_4_‐treated mice, also reduced LRAT protein expression levels in isolated (activated) hepatic stellate cells [34]. This was accompanied by reduced vitamin A autofluorescence in respective liver slices, demonstrating an association between LRAT expression levels in liver/stellate cells and vitamin A content [34]. Furthermore, a direct correlation between LRAT expression and vitamin A/retinyl ester content was demonstrated in primary rat hepatic stellate cells, using interleukin‐1 as an inflammatory cytokine that decreases LRAT expression on gene and protein level [66]. A similar downregulation of *Lrat* gene expression and reduced retinol esterification activity was also demonstrated in cultivated human liver slices, which upon cultivation showed activation of stellate cells (α‐smooth muscle actin staining) and upregulation of pro‐fibrogenic genes (*Col1a1*, *Col3a1*) [63]. In any case, an association between hepatic LRAT expression and vitamin A content is plausible since LRAT is the sole enzyme responsible for retinol esterification in liver and stellate cells and if genetically absent (knock‐out mice), virtually no retinyl esters are generated [14, 34, 66]. Consequently, given a futile retinol esterification and hydrolysis cycle, as described for triglyceride turnover [69], a downregulation of LRAT would result in reduced retinyl ester levels, providing an explanation for the observed decrease in hepatic vitamin A levels. Further, the expression of LRAT as well as the retinoid content are distinct features of hepatic stellate cells [13, 70]. Our measurements of reduced LRAT expression and retinoid content in isolated NPCs suggest that these changes observed in NPCs are due to changes specifically in hepatic stellate cells.

A very direct mode‐of‐action how hepatic vitamin A stores could be degraded is via the enzymatic hydrolysis of retinyl esters by lipases, exhibiting hydrolytic activity for retinyl esters. In the past, a number of enzymes have been demonstrated to exhibit retinyl ester hydrolase activity at neutral pH and to affect retinyl ester levels in living cells or mice [16, 17, 19, 20, 21]. These include ATGL, HSL, PNPLA3, KIAA1363, as well as some members of the carboxylesterase family, such as Ces1d, Ces1e, and Ces2c. While ATGL, HSL, and PNPLA3 are expressed in hepatocytes as well as hepatic stellate cells [18, 71, 72, 73], KIAA1363 is only detectable in hepatic stellate cells [21]. Furthermore, Ces1e (also known as Es22 or Egasyn), Ces1d, and Ces2c are predominantly expressed in hepatocytes [20, 74]. Interestingly and somehow counterintuitive, we observed mostly unchanged (ATGL, HSL, *Pnpla3*) or downregulated expression levels (*Ces1d*, *Ces1e*, *Ces2c*) of respective retinyl ester hydrolases in isolated NPCs of CCl_4_‐treated mice. These unchanged or downregulated expression levels of retinyl ester hydrolases were consistent with decreased retinyl ester hydrolase activities in respective liver lysates. Our observations on expression levels of retinyl ester hydrolases in livers of CCl_4_‐treated mice showing signs of stellate cell activation are in agreement with reports in the literature on expression levels determined in activated hepatic stellate cells. For instance, and comparable to our findings, *Atgl* gene expression has been reported to be unchanged [18] or downregulated [71], as well as gene and protein expression of HSL being downregulated in activated hepatic stellate cells [18, 71]. Gene expression of *Pnpla3* has also been reported to be decreased in rat primary activated hepatic stellate cells [18], whereas in human primary hepatic stellate cells and MASH patients in the transition from mild to severe fibrosis, the opposite was observed [75, 76]. These findings collectively suggest that upon liver injury and stellate cell activation, the expression of retinyl ester hydrolases facilitating the mobilisation/degradation of vitamin A stores is not induced. In agreement with this view of rather reduced retinyl ester degradation in CCl_4_‐treated livers, we observed reduced hepatic retinol levels, also indicative of rather reduced retinyl ester hydrolysis and retinol generation.

Numerous studies have implicated autophagy of cellular lipids (lipophagy) as a driving force for the loss of hepatic vitamin A stores upon stellate cell activation (for reviews see [36, 39, 77]). Common animal models for liver fibrosis, including CCl_4_, thioacetamide, or bile duct ligation, have reported increased autophagosome formation, with some studies also suggesting increased autophagic flux in the fibrotic livers of mice [37, 38, 39, 40, 78, 79, 80]. This increased autophagic flux is thought to also affect lipids, involving acidic hydrolysis in lysosomes (lipophagy) [38]. The main acidic hydrolytic activity for retinyl ester has been attributed to LAL [15, 23]. Similar to findings in the literature [37, 38, 39, 40, 79], we observed increased protein expression of markers for autophagosomal and lysosomal membranes in the livers of CCl_4_‐treated mice. However, hepatic protein levels of the marker protein for autophagic flux, p62, as well as the lipid marker for lysosomal membranes, bis(monoacylglycero)phosphate, were increased, arguing rather for the accumulation of autophagosomal/lysosomal membranes than increased turnover. This view is to some extent also supported by unchanged/lower mRNA expression levels of *Lipa* (LAL) as well as unchanged/lower acidic ex vivo retinyl ester hydrolase activities in liver homogenates and lysates of isolated NPCs of CCl_4_‐treated mice. Our observation suggests that lipophagy/acidic hydrolysis of retinyl ester is unchanged or rather decreased in the livers of CCl_4_‐treated mice and thus is not the driving force for decreased hepatic retinyl ester content. In line with this view, a limiting role of autophagy/lipophagy and, in this context, of LAL as the main acid hydrolase in the degradation of retinyl esters of hepatic stellate cells has been questioned already in the past by the following observations: Cell experiments with isolated primary hepatic stellate cells of LAL‐deficient mice as well as with the human hepatic stellate cell line LX‐2, using the pharmacological inhibitor Lalistat2, have established that LAL is responsible for the majority of acidic retinyl ester hydrolase activity but not required for retinyl ester breakdown. This conclusion was drawn from pulse‐chase experiments showing that, despite LAL‐knockout or pharmacological inhibition in murine and human hepatic stellate cells, similar amounts of retinyl esters were degraded in the chase period, indicating that cells were not defective in degrading retinyl esters [24]. Furthermore, also the phenotype of mice globally lacking LAL argues against a limiting role of LAL in hepatic retinyl ester degradation. Despite the massive accumulation of neutral lipids (mostly cholesterol esters and triglycerides) in various tissues including the liver, LAL‐ko mice exhibit decreased hepatic retinyl ester levels as compared to wild‐type mice, and show lipid accumulation in F4/80 positive cells (Kupffer cells) and not in GFAP‐positive cells (stellate cells) [81]. The explanations for decreased hepatic retinyl ester content of LAL‐deficient mice might be for example, due to impaired nutritional availability due to accumulation in the intestine [23], as well as decreased hepatic LRAT expression seen at advanced age [82]. However, hepatic vitamin A stores of LAL‐deficient mice upon the induction of liver fibrosis have not been investigated so far.

Another interesting finding of this study was the biphasic change in serum retinol and RBP4 levels, which were increased after 3 weeks but decreased after 6 weeks, when mice were administered CCl_4_ for 6 weeks. Under healthy conditions, serum retinol and RBP4 levels are known to be under homeostatic control, which is tightly controlled by the liver [83]. In patients with chronic liver disease, serum retinol and RBP4 levels are often lower, and the magnitude of the decrease correlates to some extent with the severity of liver disease [84, 85, 86, 87, 88, 89, 90]. However, there is a discrepancy in the literature since there are also several studies reporting the opposite effect, increased retinol and RBP4 levels [91, 92, 93]. Considering our finding of a biphasic change in serum retinol/RBP4 levels upon induction of liver damage, it suggests that the different changes in serum retinol/RBP4 levels in patients suffering from liver disease might be due to differences in the duration and severity of disease stage. Along this line, it might well be that during the initial phase of liver damage, when hepatic stellate cells get activated and LRAT expression is lost, serum retinol/RBP4 levels increase, since retinol is in surplus and not esterified in the liver. At a later, more severe stage of liver disease, serum retinol/RBP4 levels steadily decrease since liver damage compromises liver vitamin A stores (activated stellate cells), and the liver cannot maintain constant serum retinol/RBP4 levels. Furthermore, our measurements of tissue vitamin A content of CCl_4_‐treated mice indicate that, as a consequence of lower hepatic retinoid stores of damaged liver, vitamin A is redistributed to peripheral tissues in the body, such as adipose tissue and lung. Similar observations were also reported using alcohol feeding as a model for liver damage, where after a decline in hepatic retinyl ester stores, an increase in lung and white adipose tissue was observed [94].

The current study was based on the chemical induction of liver fibrosis by the hepatotoxin CCl_4_. Repeated CCl_4_ injections in mice are commonly used as a model for inducing liver fibrosis because they result in significant pathological changes in liver tissue, a short induction time, and high reproducibility [95, 96]. However, CCl_4_ also imposes certain limitations. Its toxicity is based on a non‐specific activation of hepatic cytochrome P450 enzymes to form free radicals, which damage various cellular compartments, thereby inducing liver injury [95, 96, 97]. Thus, CCl_4_‐induced liver injury does not reflect the pathophysiology of chronic liver injuries commonly seen in humans, such as alcoholic‐, diet‐, cholestasis‐, virus‐induced liver disease, or MASLD [96]. Thus, cholestasis‐ or diet‐induced liver fibrosis mouse models are more closely related to the clinical manifestation of human liver disease [96].

Together, this study investigated different pathways leading to the loss of hepatic vitamin A stores upon repeated injection of CCl_4_ into mice as a fibrosis model. We show that the decline in hepatic retinoid stores upon liver damage and stellate cell activation is mainly due to a loss of retinoids in the NPC fraction and therewith in hepatic stellate cells. In line, this loss of hepatic retinyl esters is apparently a consequence of stellate cell activation and the concomitant loss of the expression of the retinol esterifying enzyme, lecithin:retinol acyltransferase, rather than increased neutral or acidic retinyl ester hydrolysis. Moreover, we show that the loss of hepatic vitamin A stores in CCl_4_‐treated mice leads to a biphasic change in circulating retinol and RBP4 levels, which is dependent on the duration of the treatment and leads to a redistribution of retinoids to peripheral tissues.

## Author Contributions

**Carina Wagner:** conceptualisation, data curation, formal analysis, investigation, methodology, writing – original draft, review, and editing. **Ulrike Taschler and Kristina Košić:** conceptualisation, data curation, investigation, methodology, writing – review and editing. **Dominik Bulfon, Johannes Breithofer, and Clara Zitta:** data curation, investigation, methodology, writing – review and editing. **Alina Jamnik, Kim Bilweis, and Paula Horvat:** data curation, investigation, methodology. **Michael Schupp and Robert Zimmermann:** formal analysis, writing – review and editing. **Achim Lass:** conceptualisation, formal analysis, supervision, funding acquisition, methodology, project administration, writing – review and editing.

## Conflicts of Interest

The authors declare no conflicts of interest.

## Supporting information


Data S1.

Data S2.

## Data Availability

The data that support the findings of this study are listed in the article and are available from the corresponding authors upon reasonable request.
