# Supplementary material for: Reduced Esterification Rather Than Increased Hydrolysis Is Causative for Loss of Hepatic Retinoids Upon CCl4 ‐Induced Liver Injury
Source: Liver Int. 2025 Aug 6;45(9):e70213. doi: 10.1111/liv.70213 (PMC12327178; doi:10.1111/liv.70213)
Supplement: Supplementary file 1 — Data S1. [file LIV-45-0-s001.docx]

**Supporting Information**

**Reduced esterification rather than increased hydrolysis is causative for loss of hepatic retinoids upon CCl_4_-induced liver injury**

Carina Wagner^1^, Kristina Košić^1^, Dominik Bulfon^1^, Johannes Breithofer^1^, Alina Jamnik^1^, Clara Zitta^1^, Paula Horvat^1^_,_ Kim Bilweis^1^, Michael Schupp^2^, Robert Zimmermann^1,3^, Ulrike Taschler^1*^, and Achim Lass^1,3,4*^

^1^Institute of Molecular Biosciences, NAWI Graz, University of Graz, Graz, Austria; ^2^Charité Universitätsmedizin Berlin, corporate member of Freie Universität Berlin and Humboldt-Universität zu Berlin, Institute of Pharmacology, Max Rubner Center (MRC) for Cardiovascular-Metabolic-Renal Research, Berlin, Germany; ^3^BioTechMed-Graz, Graz, Austria; ^4^Field of Excellence BioHealth, University of Graz, Graz, Austria

*Correspondence: Achim Lass and Ulrike Taschler, Institute of Molecular Biosciences, University of Graz, Heinrichstraße 31/II, 8010 Graz, Austria; Phone: +43 316 380 1900; e-mail: achim.lass@uni-graz.at, [ulrike.taschler@uni-graz.at](mailto:ulrike.taschler@uni-graz.at)

**Supplementary methods:**

**Determination of *in vitro* triglyceride hydrolase activity**

Triglyceride hydrolase activity assay was performed as described previously^28^ with some modifications. In brief, 25 µl liver lysates (50 µg protein) were incubated with 25 µl substrate for 1 h at 37°C. The substrate contained triolein (1.67 mM) and [^3^H]-triolein (12.5 µCi/ml) and was emulsified with phosphatidyl choline/ inositol (PC/PI, 3/1, M/M; 190 µM). Lipids were dried under N_2_ and emulsified by sonication in 100 mM potassium phosphate buffer (pH 7.5). Then, 5% fatty acid-free BSA was added and mixed by vortexing. Reactions were terminated by the addition of 650 µl methanol:chloroform:*n*-heptane (10:9:7, v/v/v) and 200 µl 0.1 M potassium carbonate (pH 10.5). Then, samples were vigorously vortexed and centrifuged at 2,000 × *g* for 10 min. The radioactivity in 100 µl of the upper phase was determined by liquid scintillation counting. Substrate blank incubation was performed with solution A.

**Suppl. table 1**. Primer sequences used for determination of relative gene expression analysis by qPCR.

| Gene | Gene name | NCBI Accession Number | Sequences |
| --- | --- | --- | --- |
| *Adh1* | *Alcohol dehydrogenase 1* | [NM_007409.3](https://www.ncbi.nlm.nih.gov/nucleotide/NM_007409.3?report=genbank&log$=nucltop&blast_rank=2&RID=K706UFHR013) | FW: 5’-GTG ACT TGT GTG AAA CCA GGT-3’  RV: 5’-GCT ACA AAA GTT GCT TTC CGG G-3’ |
| *Adh4* | *Alcohol dehydrogenase 4* | [NM_011996.2](https://www.ncbi.nlm.nih.gov/nucleotide/NM_011996.2?report=genbank&log$=nucltop&blast_rank=4&RID=K70AAX0F016) | FW: 5’-TGG CAG TCC CCT TTG CAT T-3’  RV: 5’-ACT ACC GGG AAG AGA GCT TTC-3’ |
| *Aldh1a1* | *Aldehyde dehydrogenase 1 family member A1* | [NM_001361503.1](https://www.ncbi.nlm.nih.gov/nucleotide/NM_001361503.1?report=genbank&log$=nucltop&blast_rank=2&RID=K70CJ83K013) | FW: 5’-CTG GCT GAC TTA ATG GAG AGA GAT C-3’  RV: 5’-AGT ATG CAT TGG CAA AGA CTT TCC-3’ |
| *Abhd5* | *α/β-Hydrolase domain containing protein 5* | [NM_026179.2](https://www.ncbi.nlm.nih.gov/nucleotide/NM_026179.2?report=genbank&log$=nucltop&blast_rank=5&RID=K70WPHMJ013) | FW: 5’-TGG TGT CCC ACA TCT ACA TCA-3’  RV: 5’-CAG CGT CCA TAT TCT GTT TCC A-3’ |
| *ApoB* | *apolipo-protein B* | NM_009693.2 | FW: 5’-GCT CAA CTC AGG TTA CCG TGA-3’  RV: 5’-AGG GTG TAC TGG CAA GTT TGG-3’ |
| *α-Sma* | *Alpha-smooth muscle actin* | [NM_007392.3](https://www.ncbi.nlm.nih.gov/nucleotide/NM_007392.3?report=genbank&log$=nucltop&blast_rank=2&RID=K70H6HRD013) | FW: 5’-TCA GGG AGT AAT GGT TGG AAT G-3’  RV: 5’-TCG GCA GTA GTC ACG AAG GAA-3’ |
| *Atg7* | *autophagy related 7* | NM_001253717.2 | FW: 5’-GTT CGC CCC CTT TAA TAG TGC-3’  RV: 5’-TGA ACT CCA ACG TCA AGC GG-3’ |
| *Ces1d* | *Carboxylesterase 1D* | NM_053200.2 | FW: 5’-ATA TGG CTT TCT CTT GCT GCG-3´  RV: 5´-CCC AGG ACT TTG CCT TTA ACA GT-3´ |
| *Ces1e* | *Carboxylesterase 1E* | NM_133660.4 | FW: 5’-CCA GTG ACA GGG CAA ATA GTC-3´  RV: 5´-GTA GAC AGG ACC AGT CCA TCA TA-3´ |
| *Ces2c* | *Carboxylesterase 2C* | NM_145603.2 | FW: 5’-GCT GAA TGC TGG GTT CTT CG-3´  RV: 5´-GCT GCC TTG GAT CTG TCC TGT-3´ |
| *Col1a1* | *Collagen type 1 α 1* | NM_007742.4 | FW: 5’-CCG GCT CCT GCT CCT CCT A-3’  RV: 5’-CCA TTG TGT ATG CAG CTG ACT TC-3’ |
| *Col1a2* | *Collagen type 1 α 2* | NM_007743.3 | FW: 5'-AAG GGT GCT ACT GGA CTC CC-3'  RV: 5'-TTG TTA CCG GAT TCT CCT TTG G-3' |
| *Cyclob* | *Cyclophilin B* | [NM_011149.2](https://www.ncbi.nlm.nih.gov/nucleotide/NM_011149.2?report=genbank&log$=nucltop&blast_rank=10&RID=K71C289G016) | FW: 5′-GGC TCC GTC GTC TTC CTT TT-3′  RV: 5’-ACT CGT CCT ACA GAT TCA TCT CC-3′ |
| *Cyp26a1* | *Cytochrome P450 family 26 subfamily A member 1* | [NM_007811.2](https://www.ncbi.nlm.nih.gov/nucleotide/NM_007811.2?report=genbank&log$=nucltop&blast_rank=3&RID=K71P11G1013) | FW: 5’-TCT CCA ACC TGC ACG ATT CC-3’  RV: 5’-CGG CTG AAG GCC TGC AT-3’ |
| *Dgat1* | *diacylglycerol O-acyltransferase 1* | NM_010046.4 | FW: 5’-GTG CAC AAG TGG TGC ATC AG-3’  RV: 5’-CAG TGG GAT CTG AGC CAT CA-3’ |
| *Foxo1* | *Forkhead box protein O1* | NM_019739.3 | FW: 5’-AAG GAT AAG GGC GAC AGC AA-3´  RV: 5´-TCC ACC AAG AAC TCT TTC CA-3´ |
| *G0S2* | *G0/G1 Switch 2* | NM_008059.3 | FW: 5’-TAG TGA AGC TAT ACG TGC TGG GC-3´  RV: 5´-GGC TGG CGG CTG TGA AAG GGT-3´ |
| *Nceh1* | *Neutral cholesterol ester hydrolase 1; Kiaa1363* | [NM_178772.4](https://www.ncbi.nlm.nih.gov/nucleotide/NM_178772.3?report=genbank&log$=nucltop&blast_rank=2&RID=K7325BPV013) | FW: 5'-AAG GTC TTC TCC GAA AGT GAA GG-3'  RV: 5'-CCT CCG TGG ATA TAG ATG ACG C-3' |
| *Mttp* | *microsomal triglyceride transfer protein* | NM_001163457.2 | FW: 5‘-AGC CAG TGG GCA TAG AAA ATC-3‘  RV: 5‘-GGT CAC TTT ACA ATC CCC AGA G-3‘ |
| *Lc3* | *microtubule-associated protein 1A/1B-light chain 3* | NM_026160.5 | FW: 3’-CGG AGC TTT GAA CAA AGA GTG-5’  RV: 3’-TCT CTC ACT CTC GTA CAC TTC-5‘ |
| *Lamp1* | *lysosomal-associated membrane protein 1* | NM_001317353.1 | FW: 5'-CAG CAC TCT TTG AGG TGA AAA AC-3'  RV: 5'-ACG ATC TGA GAA CCA TTC GCA-3' |
| *Ldlr* | *low density lipoprotein receptor* | NM_010700.3 | FW: 5’-TCA GTC CCA GGC AGC GTA T-3’  RV: 5’-CTT GAT CTT GGC GGG TGT T-3’ |
| *Lipa* | *Lipase A; Lysosomal acid lipase* | [NM_021460.3](https://www.ncbi.nlm.nih.gov/nucleotide/NM_021460.3?report=genbank&log$=nucltop&blast_rank=4&RID=K735NZWJ013) | FW: 5‘-GGA ACA CTC GGT CCT GAC AG-3‘  RV: 5‘-CAC ATC AAA GCC AGC ATC CG-3‘ |
| *Lipe* | *Lipase E; Hormone-sensitive lipase* | NM_010719.5 | FW: 5’-GCT GGG CTG TCA AGC ACT GT-3’  RV: 5’-GTA ACT GGG TAG GCT GCC AT-3’ |
| *Lox* | *Lysyloxidase* | [NM_010728.4](https://www.ncbi.nlm.nih.gov/nucleotide/NM_010728.3?report=genbank&log$=nucltop&blast_rank=4&RID=K737JA8P013) | FW: 5’-TTC CAC GTA CGT CCA GAA GA-3’  RV: 5’-AGT CTC TGA CAT CCG CCC TA-3’ |
| *Lrat* | *Lecithin:retinol acyltransferase* | NM_023624.4 | FW: 5’-ACA AGG AAC GCA CTC AGA AG-3’  RV: 5’-GTC TAG GTG ATT GAC GAG GAT G-3’ |
| *Lrp1* | *Low density lipoprotein receptor-related protein 1* | NM_008512.2 | FW: 5’-ACT ATG GAT GCC CCT AAA ACT TG-3´  RV: 5´-GCA ATC TCT TTC ACC GTC ACA-3´ |
| *Pnpla2* | *Patatin-like phospholipase domain-containing protein 2* | NM_001163689.1 | FW: 5’-GAG ACC AAG TGG AAC ATC-3’  RV: 5'-GTA GAT GTG AGT GGC GTT-3' |
| *Pnpla3* | *Patatin-like phospholipase domain-containing protein 3* | [NM_054088.3](https://www.ncbi.nlm.nih.gov/nucleotide/NM_054088.3?report=genbank&log$=nucltop&blast_rank=6&RID=K73RT61D016) | FW: 5'-TCA CCT TCG TGT GCA GTC TC-3'  RV: 5'-CCT GGA GCC CGT CTC TGA T-3' |
| *p62* | *sequestosome 1* | NM_011018.3 | FW: 5'-TGT GGA ACA TGG AGG GAA GAG-3‘  RV: 5'-TGT GCC TGT GCT GGA ACT TTC-3‘ |
| *Rab7* | *ras-related protein 7* | NM_001293652.1 | FW: 5'-AGG CTT GGT GCT ACA GGA AAA-3'  RV: 5'-CTT GGC CCG GTC ATT CTT GT-3' |
| *Rarβ* | *Retinoic acid receptor beta* | NM_011243.2 | FW: 5’-CTG CTC AAT CCA TCG AGA CAC-3´  RV: 5´-CTT GTC CTG GCA AAC GAA GC-3´ |
| *Tgfβ* | *Transforming growth factor beta* | NM_011577.2 | FW: 5'-CAC CGG AGA GCC CTG GAT A-3'  RV: 5'-TGT ACA GCT GCC GCA CAC A-3' |
| *18S* | *18S rRNA* |  | FW: 5’-GTA ACC CGT TGA ACC CCA TT- 3’  RV: 5’-CCA TCC AAT CGG TAG TAG CG- 3’ |
| *Timp1* | *TIMP metallopeptidase inhibitor 1* | NM_001044384.2 | \| FW: 5‘-GCA ACT CGG ACC TGG TCA TAA-3’ \| \| --- \| \| RV: 5‘-CGG CCC GTG ATG AGA AAC T-3‘ \| |


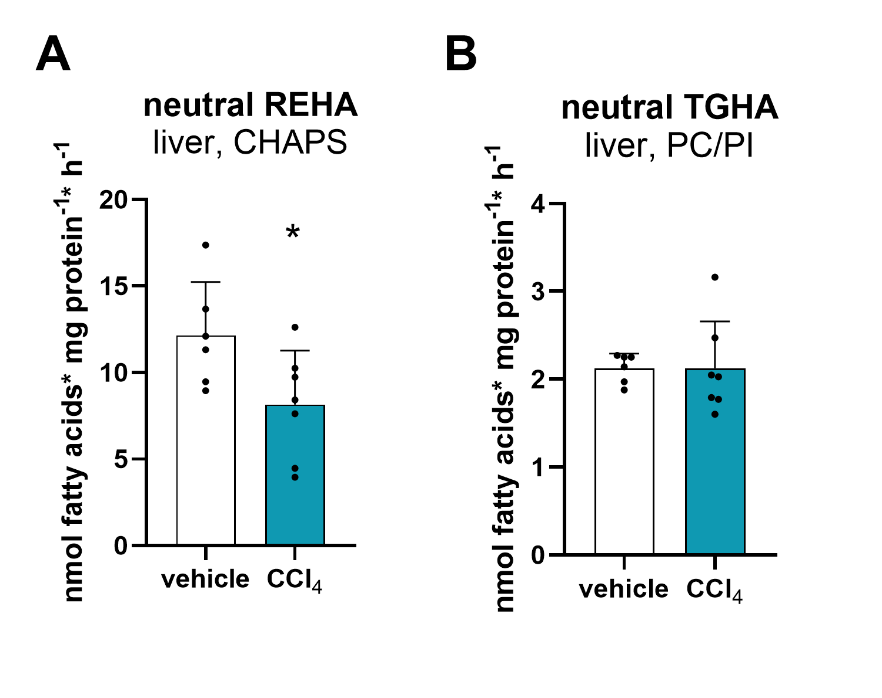


**Supplement Figure 1. *In vitro* retinyl ester hydrolase activity (REHA) and in vitro triglyceride hydrolase activity (TGHA) of liver lysates from CCl_4_- or vehicle-treated C57Bl/6J mice**. CCl_4_ (0.6 μl/g body weight, diluted in corn oil, n=7) or vehicle (corn oil, n=6) was administered (i.p.) to C57Bl/6J mice twice a week for 6 weeks. Livers were excised from *ad libitum* fed mice. For hepatic *in vitro* REHA or TGHA assay, liver lysates (1,000 x *g* supernatant) were incubated with (A) retinyl palmitate (300 μM) or (B) triolein (1.67 mM, containing [^3^H]-triolein, 12.5 µCi/ml as tracer) as substrate for 1 h at 37°C. Retinyl palmitate was emulsified with (A) CHAPS (20 mM); (B) triolein was emulsified with phosphatidyl choline/inositol (PC/PI, 3/1, 190 µM) in potassium phosphate buffer (100 mM, pH 7.5) containing 4-5% fatty acid-free BSA. (A) Fatty acid release was determined using Fujifilm NEFA-HR Assay according to manufacturer’s instructions or (B) by liquid scintillation counting. Activity assays were performed in duplicates. Data are mean +S.D. and represented as individual data points of biological replicates. Statistically significant differences were determined by Student‘s unpaired *t*-test (two-tailed; *, *p* < 0.05).
