# Supplementary material for: Reduced Esterification Rather Than Increased Hydrolysis Is Causative for Loss of Hepatic Retinoids Upon CCl4 ‐Induced Liver Injury
Source: Liver Int. 2025 Aug 6;45(9):e70213. doi: 10.1111/liv.70213 (PMC12327178; doi:10.1111/liv.70213)
Supplement: Supplementary file 2 — Data S2. [file LIV-45-0-s002.docx]

**Supporting Information**

**Reduced esterification rather than increased hydrolysis is causative for the loss of hepatic retinyl ester stores upon liver injury**

Carina Wagner^1^, Kristina Košić^1^, Dominik Bulfon^1^, Alina Jamnik^1^, Clara Zitta^1^, Paula Horvat^1^_,_ Kim Bilweis^1^, Michael Schupp^2^, Robert Zimmermann^1,3^, Ulrike Taschler^1*^, and Achim Lass^1,3,4*^

^1^Institute of Molecular Biosciences, NAWI Graz, University of Graz, Graz, Austria; ^2^Charité Universitätsmedizin Berlin, corporate member of Freie Universität Berlin and Humboldt-Universität zu Berlin, Institute of Pharmacology, Max Rubner Center for Cardiovascular-Metabolic-Renal Research, Berlin, Germany; ^3^BioTechMed-Graz, Graz, Austria; ^4^Field of Excellence BioHealth, University of Graz, Graz, Austria

*Correspondence: Achim Lass and Ulrike Taschler, Institute of Molecular Biosciences, University of Graz, Heinrichstraße 31/II, 8010 Graz, Austria; Phone: +43 316 380 1900; e-mail: [achim.lass@uni-graz.at](mailto:achim.lass@uni-graz.at) , [ulrike.taschler@uni-graz.at](mailto:ulrike.taschler@uni-graz.at)

**Suppl. Table 1**: Primer sequences used for determination of relative gene expression by qPCR

| Gene | Gene name | NCBI Accession | Sequences |
| --- | --- | --- | --- |
|  |  | Number |  |
| *Adh1* | *Alcohol dehydrogenase 1* | NM_007409.3 | FW: 5’-GTG ACT TGT GTG AAA CCA GGT-3’ |
|  |  |  | RV: 5’-GCT ACA AAA GTT GCT TTC CGG G-3’ |
| *Adh4* | *Alcohol dehydrogenase 4* | NM_011996.2 | FW: 5’-TGG CAG TCC CCT TTG CAT T-3’ |
|  |  |  | RV: 5’-ACT ACC GGG AAG AGA GCT TTC-3’ |
| *Aldh1a1* | *Aldehyde dehydrogenase 1 family member A1* | NM_001361503.1 | FW: 5’-CTG GCT GAC TTA ATG GAG AGA GAT C-3’ |
|  |  |  | RV: 5’-AGT ATG CAT TGG CAA AGA CTT TCC-3’ |
| *Abhd5* | *α/β-Hydrolase domain containing protein 5* | NM_026179.2 | FW: 5’-TGG TGT CCC ACA TCT ACA TCA-3’ |
|  |  |  | RV: 5’-CAG CGT CCA TAT TCT GTT TCC A-3’ |
| *α-Sma* | *Alpha-smooth muscle actin* | NM_007392.3 | FW: 5’-TCA GGG AGT AAT GGT TGG AAT G-3’ |
|  |  |  | RV: 5’-TCG GCA GTA GTC ACG AAG GAA-3’ |
| *Ces1d* | *Carboxylesterase 1D* | NM_053200.2 | FW: 5’-ATA TGG CTT TCT CTT GCT GCG-3´ |
|  |  |  | RV: 5´-CCC AGG ACT TTG CCT TTA ACA GT-3´ |
| *Ces1e* | *Carboxylesterase 1E* | NM_133660.4 | FW: 5’-CCA GTG ACA GGG CAA ATA GTC-3´ |
|  |  |  | RV: 5´-GTA GAC AGG ACC AGT CCA TCA TA-3´ |
| *Ces2c* | *Carboxylesterase 2C* | NM_145603.2 | FW: 5’-GCT GAA TGC TGG GTT CTT CG-3´ |
|  |  |  | RV: 5´-GCT GCC TTG GAT CTG TCC TGT-3´ |
| *Col1a1* | *Collagen type 1 α 1* | NM_007742.4 | FW: 5’-CCG GCT CCT GCT CCT CCT A-3’ |
|  |  |  | RV: 5’-CCA TTG TGT ATG CAG CTG ACT TC-3’ |
| *Col1a2* | *Collagen type 1 α 2* | NM_007743.3 | FW: 5'-AAG GGT GCT ACT GGA CTC CC-3' |
|  |  |  | RV: 5'-TTG TTA CCG GAT TCT CCT TTG G-3' |
| *Cyclob* | *Cyclophilin B* | NM_011149.2 | FW: 5′-GGC TCC GTC GTC TTC CTT TT-3′ |
|  |  |  | RV: 5’-ACT CGT CCT ACA GAT TCA TCT CC-3′ |
| *Cyp26a1* | *Cytochrome P450 family 26 subfamily A member 1* | NM_007811.2 | FW: 5’-TCT CCA ACC TGC ACG ATT CC-3’ |
|  |  |  | RV: 5’-CGG CTG AAG GCC TGC AT-3’ |
| *Foxo1* | *Forkhead box protein O1* | NM_019739.3 | FW: 5’-AAG GAT AAG GGC GAC AGC AA-3´ |
|  |  |  | RV: 5´-TCC ACC AAG AAC TCT TTC CA-3´ |
| *G0S2* | *G0/G1 Switch 2* | NM_008059.3 | FW: 5’-TAG TGA AGC TAT ACG TGC TGG GC-3´ |
|  |  |  | RV: 5´-GGC TGG CGG CTG TGA AAG GGT-3´ |
| *Ldlr* | *low density lipoprotein receptor* | NM_010700.3 | FW: 5’-TCA GTC CCA GGC AGC GTA T-3’ |
|  |  |  | \| RV: 5’-CTT GAT CTT GGC GGG TGT T-3’ \| \| --- \| |
| *Lipe* | *Lipase E; Hormone-sensitive lipase* | NM_010719.5 | FW: 5’-GCT GGG CTG TCA AGC ACT GT-3’ |
|  |  |  | RV: 5’-GTA ACT GGG TAG GCT GCC AT-3’ |
| *Lipa* | *Lipase A; Lysosomal acid lipase* | NM_021460.3 | FW: 5‘-GGA ACA CTC GGT CCT GAC AG-3‘ |
|  |  |  | RV: 5‘-CAC ATC AAA GCC AGC ATC CG-3‘ |
| *Lox* | *Lysyloxidase* | NM_010728.4 | FW: 5’-TTC CAC GTA CGT CCA GAA GA-3’ |
|  |  |  | RV: 5'-AGT CTC TGA CAT CCG CCC TA-3' |
| *Lrat* | *Lecithin:retinol acyltransferase* | NM_023624.4 | FW: 5’-ACA AGG AAC GCA CTC AGA AG-3’ |
|  |  |  | RV: 5’-GTC TAG GTG ATT GAC GAG GAT G-3’ |
| *Lrp1* | *Low density lipoprotein receptor related protein 1* | NM_008512.2 | FW: 5’-ACT ATG GAT GCC CCT AAA ACT TG-3´ |
|  |  |  | RV: 5´-GCA ATC TCT TTC ACC GTC ACA-3´ |
| *Pnpla2* | *Patatin-like phospholipase domain-containing protein 2* | NM_001163689.1 | FW: 5’-GAG ACC AAG TGG AAC ATC-3’ |
|  |  |  | RV: 5'-GTA GAT GTG AGT GGC GTT-3' |
| *Pnpla3* | *Patatin-like phospholipase domain-containing protein 3* | NM_054088.3 | FW: 5'-TCA CCT TCG TGT GCA GTC TC-3' |
|  |  |  | RV: 5'-CCT GGA GCC CGT CTC TGA T-3' |
| *Rarα* | *Retinoic acid receptor alpha* | NM_009024.2 | FW: 5’-CCA GCA CCA GCT TCC AGT CA-3’ |
|  |  |  | RV: 5’-ACT GCT GCT CTG GGT CTC GAT-3’ |
| *Rarβ* | *Retinoic acid receptor beta* | NM_011243.2 | FW: 5’-CTG CTC AAT CCA TCG AGA CAC-3´ |
|  |  |  | RV: 5´-CTT GTC CTG GCA AAC GAA GC-3´ |
| *Tgfβ* | *Transforming growth factor beta* | NM_011577.2 | FW: 5'-CAC CGG AGA GCC CTG GAT A-3' |
|  |  |  | RV: 5'-TGT ACA GCT GCC GCA CAC A-3' |
| *Timp1* | *TIMP metallopeptidase inhibitor 1* | NM_001044384.2 | FW: 5‘-GCA ACT CGG ACC TGG TCA TAA-3’ |
|  |  |  | RV: 5‘-CGG CCC GTG ATG AGA AAC T-3‘ |
